# Impact on Malaria Parasite Multiplication Rates in Infected Volunteers of the Protein-in-Adjuvant Vaccine AMA1-C1/Alhydrogel+CPG 7909

**DOI:** 10.1371/journal.pone.0022271

**Published:** 2011-07-22

**Authors:** Christopher J. A. Duncan, Susanne H. Sheehy, Katie J. Ewer, Alexander D. Douglas, Katharine A. Collins, Fenella D. Halstead, Sean C. Elias, Patrick J. Lillie, Kelly Rausch, Joan Aebig, Kazutoyo Miura, Nick J. Edwards, Ian D. Poulton, Angela Hunt-Cooke, David W. Porter, Fiona M. Thompson, Ros Rowland, Simon J. Draper, Sarah C. Gilbert, Michael P. Fay, Carole A. Long, Daming Zhu, Yimin Wu, Laura B. Martin, Charles F. Anderson, Alison M. Lawrie, Adrian V. S. Hill, Ruth D. Ellis

**Affiliations:** 1 Centre for Clinical Vaccinology and Tropical Medicine, The Jenner Institute, University of Oxford, Oxford, United Kingdom; 2 Jenner Institute Laboratories, Old Road Campus Research Building, University of Oxford, Oxford, United Kingdom; 3 National Institute of Allergy and Infectious Diseases, Bethesda, Maryland, United States; University of California Los Angeles, United States of America

## Abstract

**Background:**

Inhibition of parasite growth is a major objective of blood-stage malaria vaccines. The *in vitro* assay of parasite growth inhibitory activity (GIA) is widely used as a surrogate marker for malaria vaccine efficacy in the down-selection of candidate blood-stage vaccines. Here we report the first study to examine the relationship between *in vivo Plasmodium falciparum* growth rates and *in vitro* GIA in humans experimentally infected with blood-stage malaria.

**Methods:**

In this phase I/IIa open-label clinical trial five healthy malaria-naive volunteers were immunised with AMA1/C1-Alhydrogel+CPG 7909, and together with three unvaccinated controls were challenged by intravenous inoculation of *P. falciparum* infected erythrocytes.

**Results:**

A significant correlation was observed between parasite multiplication rate in 48 hours (PMR) and both vaccine-induced growth-inhibitory activity (Pearson r = −0.93 [95% CI: −1.0, −0.27] *P* = 0.02) and AMA1 antibody titres in the vaccine group (Pearson r = −0.93 [95% CI: −0.99, −0.25] *P* = 0.02). However immunisation failed to reduce overall mean PMR in the vaccine group in comparison to the controls (vaccinee 16 fold [95% CI: 12, 22], control 17 fold [CI: 0, 65] *P* = 0.70). Therefore no impact on pre-patent period was observed (vaccine group median 8.5 days [range 7.5–9], control group median 9 days [range 7–9]).

**Conclusions:**

Despite the first observation in human experimental malaria infection of a significant association between vaccine-induced *in vitro* growth inhibitory activity and *in vivo* parasite multiplication rate, this did not translate into any observable clinically relevant vaccine effect in this small group of volunteers.

**Trial Registration:**

ClinicalTrials.gov [NCT00984763]

## Introduction

Recent trends in the incidence of *Plasmodium falciparum* in several African countries have returned malaria eradication to the global health agenda [1]. An effective multi-stage malaria vaccine combining pre-erythrocytic and blood-stage components would significantly contribute towards eradication [2], whilst maintaining blood-stage immunity which could protect against epidemic malaria once natural immunity waned in vaccinated populations. However, despite considerable efforts, no blood stage vaccine has demonstrated clinical protection in a field trial to date (reviewed in [3], [4]).

Antibodies from malaria-immune individuals and vaccine recipients can inhibit parasite growth and invasion of erythrocytes *in vitro*, as assessed by the standardised assay of growth inhibitory activity (GIA) [5]. Assays of GIA are regarded as surrogates for candidate vaccine efficacy and are used for the down-selection of blood-stage vaccine candidates [3]. However, there are conflicting data regarding the clinical relevance of assays of GIA (reviewed in [3]), and to our knowledge no study to date has examined the relationship between *in vitro* growth inhibitory activity of vaccine-induced antibody and *in vivo* parasite growth rates in humans.

Experimental malaria infections of healthy vaccinated volunteers by mosquito bites (sporozoite challenge) or inoculation of blood-stage parasites (blood-stage challenge) provide direct evidence of candidate vaccine efficacy before progression to field trials [6]. Since the number of parasites in the infecting inoculum can be calculated after administration, blood-stage challenge allows modeling of parasite multiplication rate (PMR) for individuals [7] or groups [8] with greater accuracy [9], providing greater power to detect partial efficacy of blood-stage vaccines [9], [10]. In addition the lower starting blood-stage inocula following blood-stage challenge results in a prolonged period of sub-patent parasitaemia during which protective vaccine-induced immune responses can operate. However just thirty-one humans have been enrolled in four blood-stage challenge studies to date [9], [10], [11], [12]. Only one was a vaccine efficacy trial, in which no relationship was observed between PMR and the modest vaccine-induced antibody and T cell responses; GIA was not measured [11].

Apical membrane antigen-1 (AMA1) is a leading blood-stage vaccine candidate antigen [3], [4]. The recombinant protein vaccine AMA1-C1 is a combination of the 3D7 and FVO alleles of *P. falciparum* AMA1 [13]. AMA1-C1 adsorbed on Alhydrogel was safe and immunogenic in phase I trials [14], [15], [16] but demonstrated no protective efficacy in a phase IIb trial in children in Mali [17]. Combining AMA1-C1/Alhydrogel with the novel oligodeoxynucleotide adjuvant CPG 7909 enhanced immunogenicity in phase I trials in adults in the US [13], [18], [19] and Mali [20] and induced GIA of up to 96%. Here we report the first phase IIa efficacy trial of AMA1-C1/Alhydrogel+CPG 7909.

## Methods

### Objectives

The primary objective was to assess the relationship, if any, between *in vitro* parasite growth inhibition and parasite multiplication rate *in vivo*. The secondary objective was to attempt to detect differences in the parasite multiplication rates between the vaccinated and unvaccinated subjects. Tertiary objectives were to obtain further immunogenicity and safety data on AMA1-C1/Alhydrogel+CPG 7909 in healthy malaria-naïve adults.

### Study Design

This was a phase I/IIa open-label blood-stage malaria vaccine and challenge trial, including a control group of unvaccinated volunteers as infectivity controls. Allocation to study groups occurred at screening based on volunteer preference, as previously described [21], [22], [23]. The vaccine recipients received a single intramuscular dose of the experimental vaccine AMA1-C1/Alhydrogel+CPG 7909 on days 0 and 56, and underwent intravenous blood-stage 3D7-strain *P.falciparum* challenge 14 days after the second immunisation (on day 70). Control volunteers underwent simultaneous intravenous blood-stage challenge. Twice daily *P.falciparum* quantitative polymerase chain reaction (qPCR) samples obtained from all challenged volunteers during the intensive post-challenge follow-up period were used to estimate parasite multiplication rates per 48 hours for individual volunteers, using a published mathematical model [7]. These individual PMRs were compared to results of the *in vitro* assay of parasite growth inhibitory activity (GIA) of purified IgG obtained from individual volunteers on the day of challenge (day 70), to address the primary study outcome described above.

### Participants

The clinical trial protocol and supporting CONSORT checklist are available as Supplementary Information; see Protocol S1 and Checklist S1. The study was conducted between July 2009 and September 2010 at the Centre for Clinical Vaccinology and Tropical Medicine, University of Oxford, Oxford, UK. Screening began in July 2009, and the first volunteers were enrolled in January 2010. Healthy, malaria-naïve males and non-pregnant females aged 18–50 were invited to participate in the study. Inclusion and exclusion criteria for participation have been previously described [9], [23]. As the donor of the parasite inocula was seropositive for Epstein-Barr virus (EBV) and cytomegalovirus (CMV), EBV and CMV seropositivity was included as an inclusion criterion. Due to the potential risk of inducing autoimmunity with CPG, volunteers were also screened for anti-double stranded DNA antibodies (ds-DNA) [13]. Urine pregnancy testing of female volunteers was performed at screening, and prior to vaccination, challenge and administration of malaria treatment.

### Interventions

Vaccinated volunteers were immunised intramuscularly with AMA1-C1 (80 µg)/Alhydrogel (800 µg), mixed immediately prior to administration with 564 µg CPG 7909 formulated in saline (total volume of 0.55 ml), in alternate upper arms on days 0 and 56. Details of the manufacture and formulation of both AMA1-C1/Alhydrogel and CPG 7909 in saline, and the mixing procedure used in the clinic have been described in detail elsewhere [13], [24]. Briefly, the AMA1-C1 vaccine contains two 533 amino acid recombinant malaria proteins based on the AMA1 sequences of the FVO and 3D7 clones of *P. falciparum*. The recombinant proteins consist of the correctly folded ectodomain portions of the antigens, with the addition of a six-histidine C-terminal tag to enable protein purification, and are expressed separately in *Pichia pastoris*.

Vaccinated volunteers underwent challenge two weeks after final immunisation (day 70) together with unvaccinated control volunteers, by intravenous injection of approximately 1000 erythrocytes infected with 3D7-strain *P. falciparum* parasites. Details of the inocula preparation and administration are described below.

#### Preparation of Inocula

The origin of the blood-stage inoculum has previously been described in detail [10]. All volunteers were challenged with the same preparation of inocula. The intended inoculum was 1000 infected erythrocytes per volunteer, thawed and prepared under strict aseptic conditions. Briefly, a single vial of cryopreserved erythrocytes was thawed in a containment level III laboratory using solutions licensed for clinical use and single-use disposable consumables. A class II microbiological safety cabinet (MSC) that had not previously been used for pathogen work was used to prepare the inocula. The MSC was fumigated with formaldehyde prior to use and no contamination of the hoods was detected by settle plates. Two hundred microliters of 12% saline was added dropwise to 1 ml of thawed infected blood, left for 5 min, and an additional 10 ml of 1.6% saline added dropwise. This was centrifuged for 4 minutes at 830× g, the supernatant was removed, and 10 ml of 0.9% saline was added dropwise. The cell pellet was washed twice in 0.9% saline and resuspended in 0.9% saline in a sterile syringe for injection. The injection volume per volunteer was 5 ml containing an estimated 1000 parasitised erythrocytes based on microscopic estimates of the donor's parasite density prior to freezing. The clinical inoculum was also cultured following preparation to exclude bacterial contamination. Alternating between vaccinees and controls, all subjects were inoculated intravenously within 40 minutes of inocula preparation to maximise standardisation of the infecting dose between vaccinees and controls and to avoid potential loss of parasite viability within the syringe [11].

#### Inoculum Viability

Parasite viability was assayed by limiting dilution assay, similar to that described previously [9]. The culture period was shortened to 6 days. Quantitative PCR was used to score wells positive or negative for replicating parasites. Because the qPCR assay could also detect dead parasites, a plate of identical dilutions of inocula that had been frozen without incubation was used as a negative control. Wells were scored positive only when post-incubation parasite density was >100-fold higher than the mean of un-incubated wells at the same inoculum dilution. The number of viable parasites/ml of inoculum could then be calculated and viability expressed as a percentage of the pre-freezing microscopy-estimated parasitaemia calculated using the RBC count/ml of inoculum.

#### Post-Challenge Follow-Up

Eligibility of subjects to proceed to challenge was reviewed by the study clinician prior to inoculation. The post-challenge follow-up procedure was identical to that previously described for sporozoite challenge trials, including twice daily clinical assessment of solicited malaria symptoms, and twice daily venepuncture for thick film microscopy and qPCR [23], except that follow-up began 24 hours after inoculation [9]. Severity of clinical malaria symptomatology was assessed according to a functional scale also used for vaccine safety assessment (grade 1: minimal impact on daily activity, grade 2: interferes with daily activity, grade 3: prevents daily activity). Severity of fever (measured by oral digital thermometer) in response to malaria infection was also assessed (grade 1: 37.6°C–38°C; grade 2: >38°C–39°C; grade 3: >39°C).

Treatment was initiated in response to one of the following: the identification of a single parasite by thick blood film microscopy; the onset of significant clinical symptoms of malaria (fever >37.6°C, rigors, moderate or severe myalgia) in the presence of a positive qPCR but negative blood film; or reaching day 16 without becoming symptomatic or thick film positive. Treatment was with a standard course of artemether/lumefantrine (or chloroquine if artemether/lumefantrine was contra-indicated). Volunteers were followed-up post-treatment to observe adherence to the treatment regime and to confirm that drug cure was successful [23]. To confirm there was no transmission of blood-borne viruses from the blood-stage inocula, challenged subjects were also re-tested for HIV, HBV and HCV on day 238.

#### Safety

Vaccine adverse event data was collected throughout the study. For vaccine safety assessment vaccinated volunteers recorded their axillary temperature with a digital thermometer at least every 24 hours post-vaccination or more frequently if they had febrile symptoms. In addition, volunteers completed a seven-day symptom diary recording solicited local symptoms (injection site pain, induration, erythema, warmth and itch) and systemic symptoms (arthralgia, fatigue, feverishness, headache, malaise, myalgia, nausea) following immunisation, and any medication taken in response to these symptoms. Severity of AEs was assessed according to the functional scale described above except for injection site erythema and induration (grade 1: >0–≤20 mm, grade 2: >20–≤50 mm; grade 3 >50 mm). Laboratory safety assessment with haematology and biochemistry tests (including anti-ds DNA) occurred at days 0, 14, 28, 56, 63, 70, 84, 98, 140 and 238. In addition to guided physical examination at each timepoint, full physical examination was performed on days 28, 56, 70 and 238.

### Outcomes

#### Quantitative PCR

Blood was collected and prepared for polymerase chain reaction (PCR) as previously described [25]. Quantitative PCR (qPCR) was performed as previously described in real-time [25] except using a TaqMan® probe - 5′ FAM-AACAATTGGAGGGCAAG-NFQ-MGB 3′ (Applied Biosystems, sequence supplied by Rob Hermsen, personal communication) and 12.5 µl Universal PCR Master Mix with 5 µl sample template per reaction (25 µl final reaction volume, performed in triplicate) on an Applied Biosystems Step One Plus PCR System with quantification performed by Applied Biosystems Step One plus software v2.1. Mean parasite equivalent values below 20 per ml or with only 1 positive replicate (above 20 parasites/ml) of 3 tested were classed as negative.

#### Parasite Multiplication Rate Modelling

The *in vivo* parasite multiplication rate per 48 hour life-cycle (PMR) was estimated using a previously described model [7], [26] in which a linear time effect plus a sine-wave function of parasite growth was applied to individual volunteer's log_10_ transformed qPCR data using the equation: log(*P*) = *tm*+*a*+[*c*×sin(π*t*+*k*)], where *a* = intercept, *m* = gradient, *c* = sine wave amplitude, *k* = phase shift in sine wave, and *t* = time (days) [7], [26]. 10^(2 *m*)^ represents the 2-day (i.e., 48 hour) parasite multiplication rate. The model was constrained to the known starting log_10_ parasite inoculum (A0) at *t* = 0 as previously described [9]. These results also hold when fixing *a* = A0 and *k* = 0, or *a* = A0−*c*sin(*k*) (data not shown). 95% confidence intervals (CIs) were calculated by use of the root mean square of the SE of each parameter, which were derived by asymptotic approximation [7]. Samples obtained before the first detection of parasites were excluded from analysis, and undetectable qPCR values occurring after the first peak of parasitaemia, due to parasite cycling, were replaced with the lower limit of detection of the assay (20 parasites/mL), as previously described [7], [9]. The model was applied to all positive qPCR data from individual subjects with ≥5 positive data-points [7].

#### Growth Inhibitory Activity

Clotted blood samples (sera) were obtained on the day of enrolment (d0) and the day of parasite challenge (d70) for the assay of GIA. For the control group volunteers the day of enrolment was the same as the day of parasite challenge. GIA assays were performed as previously described [27]. IgG fractions were purified from individual sera obtained on day 0 and day 70 using protein G columns (Pierce Inc., Rockford, IL); the eluted fractions were dialyzed against RPMI 1640 (Life Technologies, Gaithersburg, MD) and concentrated with centrifugal filter devices (Millipore, Billerica, MA) to a concentration of 40 mg/ml. The purified IgGs were preadsorbed with uninfected human O-negative erythrocytes (25 µl of RBCs per 1 ml of serum sample) for 1 h to remove any anti-human erythrocyte immunoglobulins. Purified IgGs were sterilised by filtration through a 0.22-µm filter (Nalge Nunc, Rochester, NY). The GIA assay was performed on these samples using human erythrocytes parasitised with late trophozoite and schizont stages of *P. falciparum* prepared by Percoll gradient and/or 5% sorbitol treatment, with a final concentration of IgG in the test well of 10 mg/ml. Parasite growth after 40 h of culture (for 3D7 parasites) and approximately 47 h of culture (for FVO parasites) was determined by a biochemical assay specific for parasite lactate dehydrogenase, and the results were determined by the O.D._650_. The assays were performed once with samples tested in triplicate. As a positive control, rabbit AMA1-C1 IgG was used for each GIA plate. The negative control was infected RBC without any IgG. Starting parasitemia was 0.3+/−0.1%. Haematocrit was 1%, and final parasitemia was 2–3% for the negative control after 40 (3D7) or 47 (FVO) hours of culture. The results of the GIA assay with the purified IgGs were expressed as percent inhibition calculated as follows: 100−[(O.D._650_ of infected RBCs with tested IgG−O.D._650_ of normal RBCs only)/(O.D._650_ of infected RBCs without any IgG−O.D._650_ of normal RBCs only)×100]. Both 3D7 and FVO strain responses were assayed; only homologous-strain assay results are presented.

#### Enzyme-Linked Immunosorbent Assay (ELISA)

Sera for ELISA were obtained on days 0, 7, 14, 28, 56, and 63 (immunised group only), and days 70, 84, 98, 140 and 238 (both groups). ELISA was performed according to a standardised protocol which has been explained in detail previously [28]. Briefly, ELISA was performed using the clinical-grade AMA1 antigen. Sera were tested at 1∶500, 1∶10,000 and 1∶100,000 dilutions, and the dilution which gave an O.D. in the reliable range was used to calculate antibody titre. The ELISA unit value of a standard was assigned as the reciprocal of the dilution giving an O.D. 405 = 1 in a standardised assay. The absorbance of individual test samples was converted into ELISA units using a standard curve generated by serially diluting the standard in the same plate. Then, ELISA units were converted to µg/ml using a conversion factor, as reported elsewhere [29]. 3D7 (homologous strain) data to day 140 are presented.

#### Enzyme-Linked Inmmunospot (ELIspot)

Heparinised blood was obtained on days 0, 14, 56, 63 (immunised group), and days 70, 98 and 238 (both groups). Peripheral blood mononuclear cells (PBMC) were isolated as previously described [30] and IFNγ ELIspots were performed according to an established protocol, modified to use 2.5×10^5^ fresh PMBC per well [30]. The two allelic forms of the AMA-1 antigen (3D7 and FVO) were separated into nine peptide pools each containing 20mer peptides overlapping by 10 amino acids at a concentration of 5 µg/ml and assayed in duplicate. Pools 1–3 contained only 3D7-allele peptides, pools 4–6 contained common peptides shared between both alleles, and pools 7–9 contained only FVO-allele peptides. Responses were calculated by subtracting background, and plates were rejected if background responses were greater than 20 spots per well. The total allelic response (i.e. 3D7-AMA1 or FVO-AMA1) was calculated by summing responses from the relevant allele-specific and common peptide pools. Responses were expressed as spot-forming cells (SFC) per 10^6^ PBMC. 3D7 (homologous strain) data to day 98 are presented.

#### Intracellular Cytokine Staining (ICS)

PBMCs cryopreserved on the day of sampling in liquid nitrogen were thawed and incubated at 37°C overnight in complete RPMI prior to *in vitro* stimulation. 1×10^6^ PBMCs/100 µL were added to 96-well round bottom plates, and 3D7-AMA and FVO-AMA protein produced from *P. pastoris* were added to cells at a concentration of 10 µg/ml each. After 2 hours, Brefeldin A (Sigma) was added at 10 µg/ml for an additional 4 hours. Cells were surfaced stained with antibodies CD4-PerCP-Cy5.5 (RPA-T4) and CD8-PE-Cy7 (RPA-T8) (BD Biosciences). To discriminate live cells from dead, LIVE/DEAD Fixable Violet Dead Cell Stain Kit (Invitrogen) was used. For intracellular staining, cells were fixed with BD Cytofix/Cytoperm, washed, and suspended in Perm/Wash buffer for staining with antibodies CD3-APC-H7 (SK7), IFN-γ-APC (B27), IL-2-PE (MQ1-17H12), and TNF-α-FITC (mAb-11) according to the manufacture's protocol (BD Biosciences). Samples (200,000 events per sample) were acquired on a BD LSR II and analyzed using FlowJo 9.1 (Tree Star) software. Live CD3^+^ cells, CD4^+^ and CD8^+^ cells were gated to determine the frequencies of cytokine production. Samples from vaccinated volunteers on day 0 and day of challenge were analysed.

### Sample size

Sample size calculations for challenge trials must balance the need for an adequate sample with the need to reduce risk to volunteers from exposure to malaria. In most blood-stage challenge studies performed to date group sizes have been in single figures. This reflects both practical limitations on the numbers of individuals that can be recruited and challenged, and ethical constraints on challenging large numbers of volunteers with vaccines without any prior evidence of efficacy. The primary endpoint sample size calculation for this study estimated that with 5 control subjects and 12 vaccinated subjects there was greater than 80% power to demonstrate a correlation coefficient >0.5 if the true correlation coefficient was 0.7. The study was not specifically powered to assess the secondary endpoint. Although the planned sample size included 12 vaccinated volunteers and 5 controls (with a total 6 controls and 15 vaccinated volunteers to allow for drop-outs), recruitment was halted after interim primary endpoint analysis on the vaccinated volunteers (unadjusted for interim monitoring), which demonstrated that the primary endpoint had been reached after the initial challenge of 5 vaccinated volunteers and 3 controls.

To assess the primary and secondary endpoints, data from all challenged subjects for which there were adequate data were analysed. Immunogenicity and vaccine adverse event analysis for the tertiary endpoint was performed on an intention-to-treat basis incorporating all volunteers who received one or more doses of vaccine.

### Statistical Methods and Blinding

Allocation to study groups was not randomised but occurred at screening based on volunteer preference, as previously described [21], [22], [23]. Both the clinical and microscopy teams were blinded to qPCR results during challenge follow-up. Laboratory staff conducting blood-film microscopy, ELISA, ICS and GIA were also blinded to volunteer group allocation. Clinical staff could be unblinded to individual qPCR values if safety concerns arose during the challenge follow-up, although this was not required.

#### Statistical Analysis

Data was classified as skewed (ELISA, ELIspot, qPCR, ICS) or not (GIA, PMR), and the former log-transformed in Pearson's correlation coefficient (r). Groups were summarised by arithmetic (non-skewed or log transformed skewed data) or geometric means (skewed) with 95% confidence intervals (CI) or medians with inter-quartile ranges (IQR), and these were compared by t test (two sample, non-skewed), Mann-Whitney test (two sample, skewed), or Wilcoxon signed rank test (paired, skewed). Safety data analysis was mainly descriptive, however the Fisher's exact test was calculated to determine the significance of any difference in the proportions of volunteers with specific adverse events following the first and second vaccinations. All tests were two-tailed with *P*<0.05 considered significant, using STATA Release 11.0 and Graph-Pad Prism 5.0.

#### Exploratory Analysis

Time to positive qPCR or blood film was assessed using survival methods (Kaplan-Meier, log-rank test).

### Ethics

The clinical trial protocol and associated documents were approved by the Oxfordshire Research Ethics Committee (OXREC 07/H0604/137) as well as the National Institute of Allergy and Infectious Diseases (NIAID) Institutional Review Board (IRB Protocol No. 08-I-N057). Clinical Trial Authorisation was granted by the United Kingdom Medicines and Healthcare Products Regulatory Agency (MHRA 2007-005389-11). All participants gave written informed consent prior to any study procedure being undertaken. The study was conducted according to the principles of the Declaration of Helsinki (2008) and the International Conference on Harmonisation (ICH) Good Clinical Practice (GCP) guidelines. The Local Safety Committee provided safety oversight and GCP compliance was independently monitored by an external organisation (Appledown Ltd, UK).

## Results

### Participant Flow

**Figure 1:**
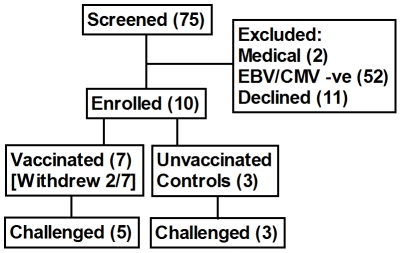
Participant Flow. Seventy-five volunteers were screened for participation, and ten volunteers were enrolled. The majority of prospective volunteers were excluded by EBV or CMV sero-negativity. Two immunised volunteers withdrew consent before the challenge due to moving from the study area, one was replaced prior to challenge.

Participant flow is summarised in Figure 1. Seventy-five potential participants were screened for inclusion. Fifty-four volunteers did not meet inclusion criteria; 52 of these had negative IgG antibody to either EBV or CMV. Six volunteers were vaccinated initially. One volunteer withdrew consent after the first vaccination after moving out of the study area, and was replaced. This replacement volunteer (V5) therefore received both vaccines with a four-week interval, whilst other volunteers received two vaccines with an eight-week interval. Both intervals were immunogenic in previous phase I trials [13], and this represented the only deviation from the study protocol during the study. Another vaccinated volunteer withdrew from the study prior to the challenge, again because they left the study area. Details of the vaccine regimens received by all volunteers are included in Supplementary Table S1.

In total five vaccinated volunteers were challenged together with three unvaccinated control volunteers.

### Recruitment

Recruitment began in July 2009 and continued until March 2010. The first volunteers were vaccinated in January 2010 and the challenge occurred in April 2010. The final study visits occurred in September 2010.

### Baseline data

The baseline demographic details of the participants in the vaccine and control groups are included in Table 1. The groups were similar in distribution for both age (vaccinees median 28 (range: 22–45), controls median 27 (range 25–29) and gender (male∶ female ratio in vaccinees 4∶ 3; controls 1∶ 2). Inoculum viability was 25% of the pre-freeze parasitemia, therefore the actual inoculum delivered was 250 viable parasites per volunteer.

**Table 1:** Volunteer Demographics.

Characteristics	Vaccine Group (n = 7)	Control Group (n = 3)
Male (%)*	4 (57)	1 (33)
Median age (range)†	28 (22–45)	27 (25–29)
Median time to inoculation in minutes (IQR)† μ	4 (0.5–7.5)	4 (0–4)

No significant differences were identified between the groups using

* Fisher's exact test (*P* = 1.0) and

† Mann-Whitney test (*P* = 0.83).

μ Applies only to the 5/7 vaccinated volunteers who underwent challenge. IQR = interquartile range.

### Primary Outcome

**Figure 2:**
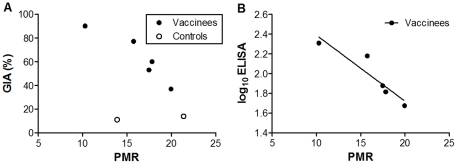
Vaccine-Induced *in vitro* Growth Inhibitory Activity (GIA) and Antibody Titre Correlates with *in vivo* Parasite Multiplication Rate (PMR). (•) Represents immunised volunteers and (○) represents control volunteers. All analyses are two-tailed Pearson correlation coefficients. Assays of GIA and ELISA (both 3D7-AMA1) were performed once in triplicate on day of challenge samples. GIA is expressed as percent inhibition calculated as follows: 100−[(O.D._650_ of infected RBCs with tested IgG−O.D._650_ of normal RBCs only)/(O.D._650_ of infected RBCs without any IgG−O.D._650_ of normal RBCs only)×100]. ELISA units are log_10_ µg/ml. Parasite multiplication rate per 48 hours was modelled from qPCR data. **A.** Correlation between vaccine-induced GIA on day of challenge and 48-hour PMR (r = −0.93 [95% CI: −1.0, −0.27] *P* = 0.02). When all volunteers (vaccinated and control) were examined together there was a trend towards an association (r = −0.61 [95% CI: −0.94, 0.27] *P* = 0.15). **B.** Correlation between log_10_ transformed 3D7-AMA1 ELISA (µg/ml) and 48-hour PMR (r = −0.93 [95% CI: −0.99, −0.25] *P* = 0.02).

Only three positive qPCR data points were available for one control volunteer (C1), insufficient for accurate estimation of PMR for this individual. This volunteer is therefore excluded from primary and secondary outcome analysis. There was a significant inverse correlation between PMR and homologous-strain (3D7) GIA on day of challenge (day 70) in the vaccine recipients (r = −0.93 [95% CI: −1.0, −0.27] *P* = 0.02, Figure 2A). This was also observed for log_10_ ELISA against homologous-strain (3D7) AMA1 on day of challenge (day 70) (r = −0.93 [95% CI: −1.0, −0.25] *P* = 0.02, Figure 2B). When immunised and control volunteers were analysed together the GIA correlation became non-significant (r = −0.61 [95% CI: −0.94, 0.27] *P* = 0.15, Figure 2A). No correlation was observed with PMR and heterologous-strain (FVO) GIA and log_10_ ELISA responses (data not shown).

### Secondary Outcome

**Figure 3:**
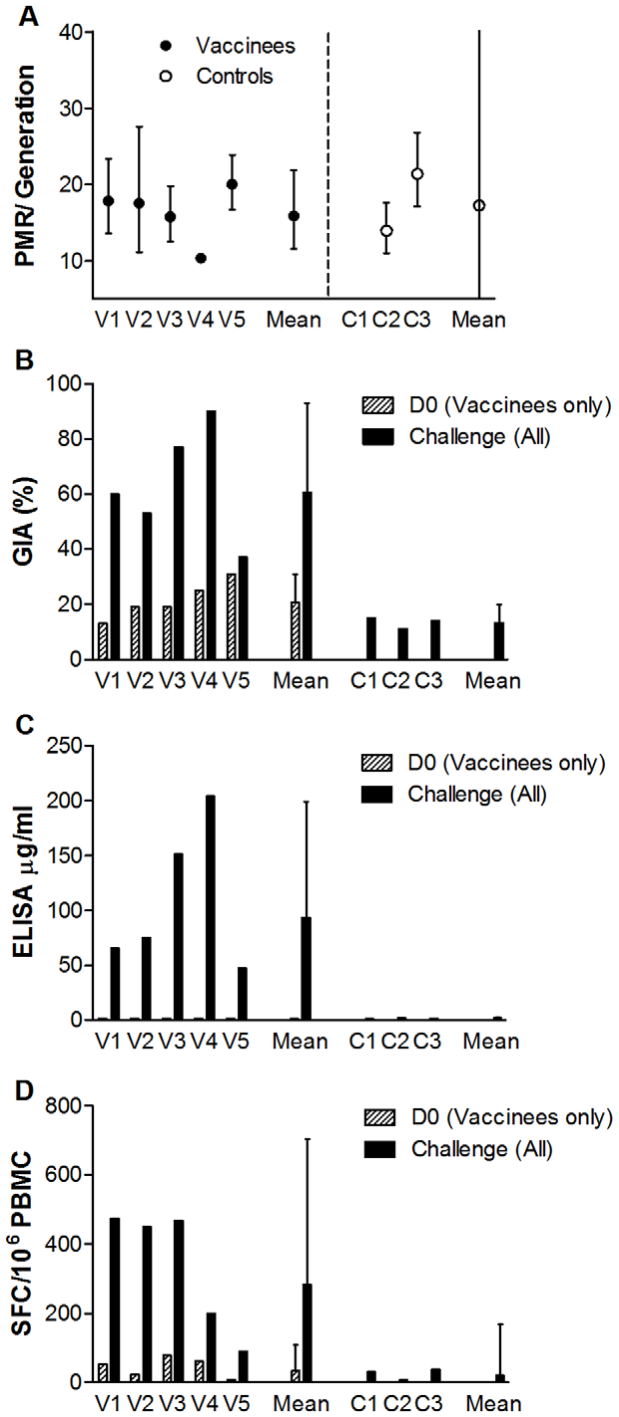
Individual Subject Parasite Multiplication Rates and Immunological Measures at Day of Challenge. (•) Represents immunised volunteers and (○) represents control volunteers. All panels display means and error bars represent 95% confidence intervals. Assays of GIA and ELISA were performed once in triplicate. ELIspot assays were performed once in duplicate. GIA is expressed as percent inhibition calculated as follows: 100−[(O.D._650_ of infected RBCs with tested IgG−O.D._650_ of normal RBCs only)/(O.D._650_ of infected RBCs without any IgG−O.D._650_ of normal RBCs only)×100]. ELISA units are µg/ml, ELIspot units are IFN-γ spot forming colonies (SFCs) per 10^6^ PBMCs. Parasite multiplication rate per 48-hours was modelled from qPCR data. **A.** 48-hour parasite multiplication rates (PMR) for individuals and arithmetic mean 48-hour PMR for the group. PMR for volunteer C1 could not be accurately modelled as there were only three qPCR data-points [7]. Arithmetic mean PMRs were not significantly different (vaccine 16-fold [95% CI: 12–22] (n = 5), control 17-fold [95% CI: 0–65] (n = 2) *P* = 0.70, t test). **B.** Individual and group mean percentage GIA. There were similar levels of detectable GIA in all volunteers at enrollment (d0 for immunised group, day of challenge for control group); mean vaccine group 21% [95% CI: 13–30] (n = 5), control group 13% [95% CI: 8–19] (n = 3) *P* = 0.10, t test. **C.** Geometric mean antibody ELISA (µg/ml) and D. geometric mean T cell ELIspot responses (IFN-γ SFC/10^6^ PMBC) to 3D7-AMA1 at day 0 (immunised group) and day of challenge (all groups, the first assessment for controls was day of challenge). All immunology endpoints were significantly higher in vaccinees than controls at challenge (GIA *P*<0.01 t test; ELIspot *P* = 0.04 Mann-Whitney; ELISA *P* = 0.04 Mann-Whitney).

Assessment of secondary outcome was limited by having only two control volunteers in which PMR could be accurately modelled. No significant difference was observed in mean PMR between the vaccine and control groups (vaccine group 16-fold [95% CI: 12–22], control group 17-fold [95% CI: 0–65], *P* = 0.70 t test, Figure 3A). The parasite multiplication rates in 48 hours (with 95% CIs) for individual vaccinated volunteers were: V1: 17.8 fold (13.6–23.3); V2: 17.5 fold (11.1–27.6); V3: 15.7 fold (12.5–19.8); V4: 10.3 fold (10.1–10.4); V5: 20.0 fold (16.7–23.9). For individual control volunteers the PMR was C2: 13.9 fold (11.0–17.6), and C3: 21.4 fold (17.1–26.8), Figure 3A. The modelling strategy fitted the data well. The mean R^2^ value was 0.93 for vaccinated subjects and 0.92 for controls with a mean of 6.6 PCR data points per volunteer. Raw qPCR data are summarised in Supplementary Table S2.

### Tertiary Outcomes

#### Antibody Immunogenicity

On the day of challenge, mean percentage vaccine-induced GIA was significantly greater in the vaccine group (63% [95% CI: 38–90]) than the control group (13% [95% CI: 9–19] *P*<0.01, t test Figure 3B). GIA was maintained but not boosted at 28 days post-challenge (61% [95% CI: 17–100]), and did not increase significantly post-challenge in the control group (data not shown).

**Figure 4:**
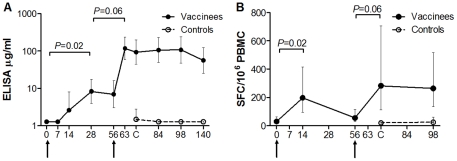
Timecourse of Homologous (3D7) Strain Antibody and T Cell Responses. (•) Represents immunised volunteers and (○) represents control volunteers. Numbers on ‘x’ axes represent days of follow-up. Arrows represent immunisations. Geometric mean 3D7-strain AMA1 antibody responses by ELISA (µg/ml) (A) and *ex vivo* 3D7-strain IFN-γ ELIspot (SFC/10^6^ PMBC) (B) for immunised volunteers (n = 7) and controls (n = 3) are presented. ELISA assays were performed once in triplicate. ELIspot assays were performed once in duplicate. Statistical comparisons are with the two-tailed Wilcoxon signed rank test. **A.** A significant increase in 3D7-strain AMA1 antibody responses by ELISA (µg/ml) was observed for first immunisation (n = 7). Responses were non-significantly boosted by the second immunisation in challenged volunteers (n = 5) and were maintained at day 140. No significant increase in detectable response was identified in the control volunteers post-challenge. **B.** A significant increase in *ex vivo* 3D7-strain IFN-γ ELIspot (SFC/10^6^ PMBC) response was observed following the first immunisation in all volunteers (n = 7). Responses were non-significantly boosted by the second immunisation in challenged volunteers (n = 5). No significant increase in detectable response was observed in control volunteers post-challenge.

A significant increase in geometric mean 3D7-strain antibody response measured by ELISA (µg/mL) was observed after the first immunisation (n = 7) (d28: 8.3 µg/mL [95% CI: 4.0–17.5] vs d0: 1.27 µg/mL [range 1.27–1.27], *P* = 0.02 Wilcoxon signed rank test, Figure 4A). Responses were non-significantly boosted by the second immunisation in challenged volunteers (n = 5) (d63: 117.4 µg/mL [95% CI: 58.9–233.9] vs d28, *P* = 0.06 Wilcoxon signed rank test, Figure 4A), and were maintained but not boosted by challenge. ELISA titre was significantly higher in vaccinated volunteers at day of challenge (geometric mean vaccine group 93.6 µg/ml [95% CI: 43.9–199.3], control group 1.5 µg/ml [95% CI: 0.8–2.8] *P* = 0.04, Mann-Whitney test, Figure 3C). No significant increase in ELISA titre was identified in the control volunteers (n = 3) post-challenge.

As we have previously observed, 3D7 GIA and 3D7 log_10_ ELISA titres correlated strongly (r = 0.97, *P*<0.01), as did 3D7 and FVO log_10_ ELISA titres (r = 0.90, *P* = 0.03) [13].

#### Cellular Immunogenicity

A significant increase in geometric mean T cell response measured by *ex vivo* IFN-γ ELIspot (spot-forming colonies (SFC) per 10^6^ PBMCs) was observed following the first immunisation in all volunteers (n = 7) (d14: 197.8 IFN-γ SFC/10^6^ PBMCs [95% CI: 94.2–415.7]) vs d0: 30.1 IFN-γ SFC/10^6^ PBMCs [95% CI: 14.3–62.0], *P* = 0.02 Wilcoxon signed rank test, Figure 4B). These responses contracted non-significantly by day 56 after the first immunisation (geometric mean 63.6 IFNγ SFC/10^6^ PBMCs [95% CI: 27.3–147.9]; P = 0.06, Wilcoxon signed-rank test), and were non-significantly boosted by the second immunisation in challenged volunteers (n = 5) (d70 geometric mean 282.4 IFN-γ SFC/10^6^ PBMCs [95% CI: 113.3–704.3] *P* = 0.06 Wilcoxon signed rank test, Figure 4B). T cell responses by ELIspot were significantly higher in vaccinated volunteers at day of challenge (geometric mean vaccine group 282.4 IFN-γ SFC/10^6^ PBMCs [95% CI: 113.3–704.3], control group 20.9 IFN-γ SFC/10^6^ PBMCs [95% CI: 8.0–38.0] *P* = 0.04, Mann-Whitney test, Figure 3D). No significant increase in detectable response was observed in control volunteers post-challenge, Figure 4B.

**Figure 5:**
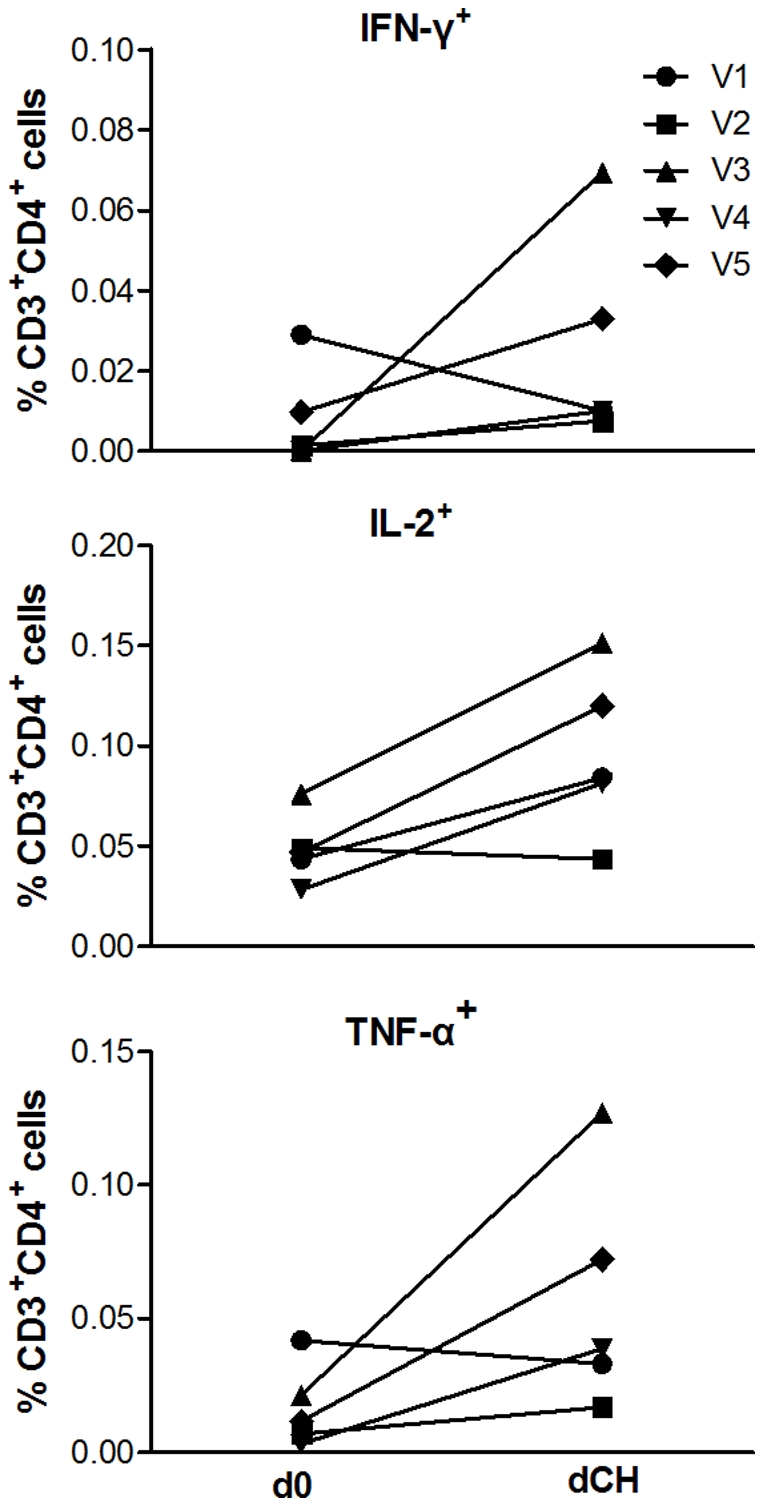
Intracellular Cytokine Staining. AMA1-C1 protein-stimulated live CD3^+^ CD4^+^ T cells positive for the Th1 cytokines IFN-γ TNF-α and IL-2 assayed on cryopreserved PBMCs obtained on the day of enrollment (d0) and day of challenge (dCH) from vaccinated and challenged volunteers (n = 5). Statistical comparisons are with the two-tailed Wilcoxon signed rank test. There was a non-significant increase in median percentage of live CD3^+^ CD4^+^ cells positive for TNF-α, IFN-γ and IL-2 (TNF-α d0: 0.003% [range 0.002–0.014], dCH: 0.009% [range 0.0–0.035], *P* = 0.31; IFN-γ d0: 0.0% [range 0.0–0.020], dCH: 0.010% [0.0–0.069], *P* = 0.58; IL-2 d0: 0.036% [range 0.022–0.061], dCH: 0.051% [range 0.035–0.072], *P* = 0.13 Wilcoxon signed rank test).

The phenotype of the vaccine-induced T cell responses was predominantly CD4^+^ by flow cytometry (data not shown). On the day of challenge (dCH) AMA1-C1 antigen-stimulated Th1 responses were detected in all vaccinated volunteers (n = 5) and multifunctional responses were detected. There was a non-significant trend to an increase in the frequency of live CD3^+^ CD4^+^ T cells positive for TNF-α, IFN-γ and IL-2 after vaccination (TNF-α d0: 0.003% [range 0.002–0.014], dCH: 0.009% [range 0.0–0.035], *P* = 0.31; IFN-γ d0: 0.0% [range 0.0–0.020], dCH: 0.010% [0.0–0.069], *P* = 0.58; IL-2 d0: 0.036% [range 0.022–0.061], dCH: 0.051% [range 0.035–0.072], *P* = 0.13 Wilcoxon signed rank test, Figure 5). There was no significant correlation between T cell responses on the day of challenge, measured by either ELIspot or ICS, and *in vivo* parasite multiplication (data not shown).

### Adverse Events

**Figure 6:**
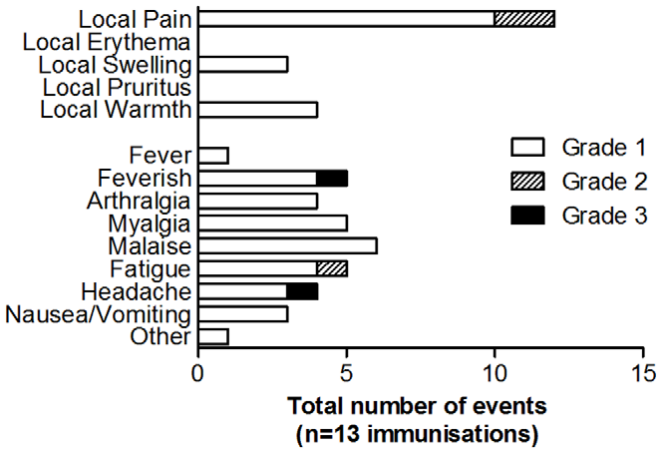
Adverse Events. All solicited and unsolicited adverse events post-vaccination considered possibly, probably or definitely vaccine-related up to day 140. One volunteer experienced a grade 3 headache and rigors on the evening of the first dose (day 0) which required oral analgesia and resulted in a missed day of work. The rigors resolved within several hours and the headache reduced in intensity within 24 hours, and resolved on day 2. ‘Other’ refers to transient injection-site discomfort relating to minor trauma 5 days after vaccination.

No unexpected or serious adverse events (AEs) occurred. Vaccine-related AEs occurred at a similar frequency following both vaccinations (Supplementary Table S3). The majority of AEs were grade 1 in severity (Figure 6). Overall, the median duration of all injection-site AEs was 3 days (IQR: 2–3.75). There was no significant difference in the duration of local or systemic AEs between the first or second doses (local *P* = 0.14; systemic *P* = 0.12, Mann Whitney test). The only vaccine-related laboratory abnormality was transient grade 1 leucopenia in a single subject following the first vaccination, which is expected with CPG 7909 [13]. Double-stranded DNA antibodies were not observed in any volunteers.

### Ancillary analyses

#### Challenge Endpoints

**Figure 7:**
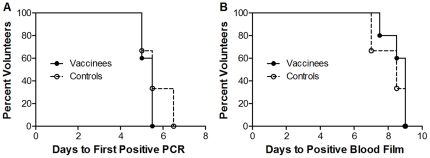
Kaplan-Meier Survival Analysis of Time to Parasitaemia. Survival analysis of **A.** time to parasitaemia by thick blood film microscopy (*P* = 0.45 log-rank test), vaccine group (bold line) median 8.5 days (range 7.5–9), control group median 9 days (range 7–9) **B.** Time to first positive qPCR value (*P* = 0.40 log-rank test), vaccine group median 5.5 days (range 5–5.5), control group median 5.5 days (range 5–6.5).

**Figure 8:**
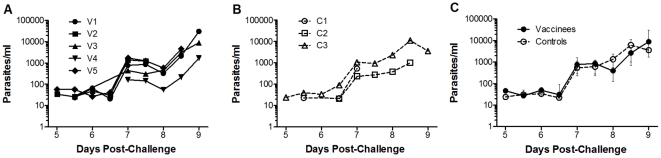
Quantitative PCR. Individual qPCR data (parasites/ml) for **A.** immunised and **B.** control volunteers. **C.** Median qPCR data (with interquartile ranges) for vaccine and control groups. No significant differences were observed between the groups at any time-point (data not shown). qPCR was performed once in triplicate at each time-point.

All volunteers were inoculated within 40 minutes of inocula preparation. All volunteers developed microscopy positive parasitaemia on thick-film by day 9 post-challenge (range 7–9). Although not surprising for a small sample, there was no significant difference in pre-patent period between the vaccine and control groups by survival analysis (vaccine group median 8.5 days (range 7.5–9), control group median 9 days (range 7–9) *P* = 0.45 log-rank test, see Figure 7A). There was also no significant difference in days to first positive PCR between the vaccine and control groups by survival analysis (vaccine group median 5.5 days (range 5–5.5), control group median 5.5 days (range 5–6.5) *P* = 0.40 log-rank test, see Figure 7B). Individual volunteer qPCR values are displayed in Figure 8A (vaccine group) and Figure 8B (control group). Parasite density at diagnosis (vaccine group median 4602 p/ml [IQR: 1472–19632], control group median 3613 p/ml [IQR: 543–11402]) was not significantly different (*P* = 0.79 Mann-Whitney test, Figure 8C). Only 2/8 volunteers developed a malaria symptom (grade 1 myalgia in 2/8, feverishness in 1/8) at the time of blood-film diagnosis. After treatment initiation six volunteers developed minor grade 1 malaria symptoms. None had objective fever. There were no challenge-related laboratory abnormalities.

## Discussion

We observed a significant inverse relationship between vaccine-induced GIA and parasite multiplication rate in vaccinated subjects. This is an important observation in a small sample, and needs to be confirmed in larger studies. However, there was no evidence to suggest that the magnitude of the vaccine-induced effect was sufficient to impact overall PMR in the vaccinated volunteers, and there was consequently no effect on pre-patent period. However the small sample size, particularly that of the control group, limited the statistical power to assess differences in PMR between the groups.

GIA induced in most vaccinated volunteers may have been insufficient to significantly reduce PMR in comparison to unvaccinated controls. Although the volunteer with 90% *in vitro* growth-inhibitory activity had the lowest parasite multiplication rate (10-fold), in most volunteers the GIA did not reach this level. Other adjuvanted AMA1 vaccines have induced similar levels of GIA without a significant impact on estimated PMR, albeit after sporozoite challenge [31]. Modelled estimates suggest much greater antibody levels are required to control *in vivo* parasite growth [8]. Non-human primate studies have shown that very high antibody titers and *in vitro* growth inhibition of >70% (using serum, not purified IgG) are needed for protection with AMA1 [32]. Similar results in primates have been reported for another blood-stage antigen, MSP1 [33]. A recent study indicates the pre-patent PMR in semi-immune Gambian adults is significantly lower than has ever been achieved by a blood-stage vaccine candidate in humans [34].

As well as antibodies [35], other immune responses are likely to be involved in determining *in vivo* PMR, including, cytokines [36], T effector [12] and T regulatory cells [36] and antibody-dependent cellular inhibition (ADCI) by monocytes [37], [38] and neutrophils [39]. We observed a clear relationship between homologous-strain log_10_ ELISA titre and PMR in vaccinees but there was no relationship between PMR and the modest vaccine-induced CD4^+^ T cell responses measured by *ex-vivo* IFN-γ ELIspot or ICS. This may reflect the insufficient magnitude of the T cell response, lack of statistical power to detect such a relationship, analysis of the non-protective T cell phenotype, or the absence of an association.

The association between GIA and PMR in vaccinees reported in this study provides some support for a protective role of very high levels of GIA-inducing antibodies, but this result need not imply causation. A recent meta-analysis of prospective sero-epidemiological studies demonstrated an association between IgG to merozoite proteins and reduced clinical malaria [35]. Fewer studies have prospectively assessed functional antibody responses such as GIA [3], [35]. Most of these studies suggest that antibody demonstrating inhibitory activity *in vitro* contributes to a reduced risk of clinical malaria [5], [40], [41], although some do not [42] (reviewed in [3]). The data are similarly conflicting in numerous animal models [32], [33], [43], suggesting that multiple potential immune effector mechanisms may operate in humans (reviewed in [3]). Data from a sporozoite challenge trial of an AMA1-containing multi-stage virosomal vaccine demonstrated significant reduction in PMR without detectable GIA or cellular responses [23]. However, there was also a trend to reduced liver-emerging parasites. A similar significant reduction in liver-emerging parasites was observed with the adjuvanted AMA1 vaccine discussed above [31], which may have also induced strain-specific efficacy in a field trial in Malian children [Ouattara A., Takala-Harrison S. et al. Allele-Specific Efficacy of the Monovalent Apical Membrane Antigen 1 (AMA1) Malaria Vaccine FMP2.1/AS02A, American Society of Tropical Medicine and Hygiene, November 2010, Abstract #803]. In these studies it is therefore difficult to rule-out the possibility that PMR could have been influenced by immune responses to sporozoites, liver-stage parasites or liver-emerging merozoites, all of which express AMA1 [31]. An inherent limitation of the blood-stage challenge model is thus an inability to detect any pre-erythrocytic vaccine efficacy. Another limitation specific to this inoculum is the requirement for volunteer EBV and CMV seropositivity, which adversely impacted recruitment in this study.

There was an unexpectedly low frequency of clinical malaria symptoms pre-diagnosis (2/8 subjects) in this study in comparison to published data on the clinical features of experimental malaria in healthy volunteers following sporozoite challenge [44]. Parasite density at microscopic diagnosis (geometric-mean 4012 p/ml) was similar to that recorded in sporozoite-challenged control volunteers in a recent study (geometric-mean 4030 p/ml), 10/12 of whom were symptomatic at diagnosis [Ewer K, O'Hara GA, Duncan CJA *et al.*, submitted], suggesting potential attenuated pathogenicity of the blood-stage challenge parasites. Differential expression and rate of switching of expressed *var* genes by parasites in this inoculum after blood-stage passage has been reported, which could explain the reduction in clinical symptoms observed [45].

This is the first trial in humans to explore the relationship between vaccine-induced inhibitory antibodies and *in vivo* parasite growth rates following experimental blood-stage malaria infection. It increases by more than a quarter the total number of volunteers who have been experimentally infected with this inoculum, and is only the second vaccine efficacy study using this model. As the first Phase IIa challenge trial of AMA1-C1/Alhydrogel+CPG 7909, it contributes further safety and immunogenicity data on this protein-adjuvant combination. However, there is insufficient evidence to support future phase IIb clinical trials of this vaccine formulation in the immunisation regimen assessed here. While the blood-stage challenge model has limitations, the use of challenge studies (both blood-stage and sporozoite) should greatly speed the clinical development of blood-stage vaccines, allowing early demonstration of possible benefit and rational down-selection of vaccine candidates prior to field trials.

## Supporting Information

Table S1 **Vaccine Regimens.** V5 was immunised to replace V6 who withdrew from the study on day 28. V5 therefore received two immunisations 28 days apart, but this dose interval did not impact on vaccine immunogenicity [since fold-increase in ELISA titre (µg/mL) following the second immunisation for V5 was similar to V1–V4 (data not shown)]. Volunteers V1–V5 were challenged simultaneously 14 days after the final immunisation.
(DOC)

Table S2 **Raw qPCR Dataset.** D = day post-challenge, V = vaccinated subject, C = control. Bold text = qPCR on day of blood film diagnosis.
(DOC)

Table S3 **Solicited and Unsolicited Adverse Events (AEs) Post-Vaccination with AMA1-C1/Alhydrogel+CPG 7909.** The maximum severity of any AE experienced by the volunteer is recorded. Overall percentage of AEs experienced by volunteers after either dose is summarised in final column. Some AEs were reported by the same volunteer after both immunisations. ^*^Both grade 3 systemic AEs occurred simultaneously in the same volunteer. ^†^Recurrent minor transient discomfort at injection-site day 5 following dose 1. No significant differences in proportion of volunteers experiencing AEs between dose 1 and dose 2 were identified by Fisher's exact test.
(DOC)

Protocol S1 **Trial Protocol.**
(PDF)

Checklist S1 **CONSORT Checklist.**
(DOC)
