# Supplementary material for: Impact on Malaria Parasite Multiplication Rates in Infected Volunteers of the Protein-in-Adjuvant Vaccine AMA1-C1/Alhydrogel+CPG 7909
Source: PLoS One. 2011 Jul 22;6(7):e22271. doi: 10.1371/journal.pone.0022271 (PMC3142129; doi:10.1371/journal.pone.0022271)
Supplement: Table S1 — Vaccine Regimens. V5 was immunised to replace V6 who withdrew from the study on day 28. V5 therefore received two immunisations 28 days apart, but this dose interval did not impact on vaccine immunogenicity [since fold-increase in ELISA titre (µg/mL) following the second immunisation for V5 was similar to V1–V4 (data not shown)]. Volunteers V1–V5 were challenged simultaneously 14 days after the final immunisation. (DOC) [file pone.0022271.s001.doc]

| **Volunteer number** | **Second immunisation (days from first immunisation)** | **Timing of withdrawl** | **Reason for withdrawl** | **Challenged** |
| --- | --- | --- | --- | --- |
| V1 | d56 | - | - | Yes |
| V2 | d56 | - | - | Yes |
| V3 | d56 | - | - | Yes |
| V4 | d56 | - | - | Yes |
| V5 | d28 | - | - | Yes |
| V6 | - | d28 | Moved from study area | No |
| V7 | d56 | d63 | Moved from study area | No |
