# Supplementary material for: Impact on Malaria Parasite Multiplication Rates in Infected Volunteers of the Protein-in-Adjuvant Vaccine AMA1-C1/Alhydrogel+CPG 7909
Source: PLoS One. 2011 Jul 22;6(7):e22271. doi: 10.1371/journal.pone.0022271 (PMC3142129; doi:10.1371/journal.pone.0022271)
Supplement: Table S2 — Raw qPCR Dataset. D = day post-challenge, V = vaccinated subject, C = control. Bold text = qPCR on day of blood film diagnosis. (DOC) [file pone.0022271.s002.doc]

| **Vol** | **D1** | **D2** | **D2.5** | **D3** | **D3.5** | **D4** | **D4.5** | **D5** | **D5.5** | **D6** | **D6.5** | **D7** | **D7.5** | **D8** | **D8.5** | **D9** |
| --- | --- | --- | --- | --- | --- | --- | --- | --- | --- | --- | --- | --- | --- | --- | --- | --- |
| **V1** | 0.0 | 0.0 | 0.0 | 0.0 | 0.0 | 0.0 | 0.0 | 0.0 | 24.0 | 66.0 | 21.0 | 777.0 | 869.0 | 329.0 | 2139.0 | **30332.0** |
| **V2** | 0.0 | 0.0 | 0.0 | 0.0 | 0.0 | 0.0 | 0.0 | 35.0 | 26.0 | 49.0 | 31.0 | 1402.0 | **1226.0** |  |  |  |
| **V3** | 0.0 | 0.0 | 0.0 | 0.0 | 0.0 | 0.0 | 0.0 | 0.0 | 29.0 | 0.0 | 0.0 | 444.0 | 310.0 | 466.0 | 3199.0 | **8932.0** |
| **V4** | 0.0 | 0.0 | 0.0 | 0.0 | 0.0 | 0.0 | 0.0 | 0.0 | 0.0 | 0.0 | 0.0 | 164.0 | 146.0 | 56.0 | 216.0 | **1718.0** |
| **V5** | 0.0 | 0.0 | 0.0 | 0.0 | 0.0 | 0.0 | 0.0 | 58.0 | 57.0 | 27.0 | 42.0 | 1735.0 | 1260.0 | 591.0 | **4602.0** |  |
| **C1** | 0.0 | 0.0 | 0.0 | 0.0 | 0.0 | 0.0 | 0.0 | 0.0 | 23.0 | 0.0 | 22.0 | **543.0** |  |  |  |  |
| **C2** | 0.0 | 0.0 | 0.0 | 0.0 | 0.0 | 0.0 | 0.0 | 0.0 | 0.0 | 0.0 | 21.0 | 233.0 | 279.0 | 381.0 | 1006.0 | **3613.0** |
| **C3** | 0.0 | 0.0 | 0.0 | 0.0 | 0.0 | 0.0 | 0.0 | 24.0 | 40.0 | 34.0 | 90.0 | 1085.0 | 937.0 | 2343.0 | **11402.0** |  |
