# Supplementary material for: Impact on Malaria Parasite Multiplication Rates in Infected Volunteers of the Protein-in-Adjuvant Vaccine AMA1-C1/Alhydrogel+CPG 7909
Source: PLoS One. 2011 Jul 22;6(7):e22271. doi: 10.1371/journal.pone.0022271 (PMC3142129; doi:10.1371/journal.pone.0022271)
Supplement: Table S3 — Solicited and Unsolicited Adverse Events (AEs) Post-Vaccination with AMA1-C1/Alhydrogel+CPG 7909. The maximum severity of any AE experienced by the volunteer is recorded. Overall percentage of AEs experienced by volunteers after either dose is summarised in final column. Some AEs were reported by the same volunteer after both immunisations. *Both grade 3 systemic AEs occurred simultaneously in the same volunteer. †Recurrent minor transient discomfort at injection-site day 5 following dose 1. No significant differences in proportion of volunteers experiencing AEs between dose 1 and dose 2 were identified by Fisher's exact test. (DOC) [file pone.0022271.s003.doc]

| **Adverse Event** | **Dose 1 (n = 7)**  N. of volunteers reporting by severity | | | **Dose 2 (n = 6)**  N. of volunteers reporting by severity | | | **All (n = 13 doses)**  N. (%) of volunteers reporting AE at least once by severity | | |
| --- | --- | --- | --- | --- | --- | --- | --- | --- | --- |
|  | *Severity* (grade) | | | *Severity* (grade) | | | *Severity* (grade) | | |
|  | 1 | 2 | 3 | 1 | 2 | 3 | 1 | 2 | 3 |
| Erythema | 0 | 0 | 0 | 0 | 0 | 0 | 0 | 0 | 0 |
| Pain | 6 | 0 | 0 | 2 | 2 | 0 | 4 (57) | 2 (29) | 0 |
| Swelling | 1 | 0 | 0 | 1 | 0 | 0 | 2 (29) | 0 | 0 |
| Warmth | 1 | 0 | 0 | 3 | 0 | 0 | 4 (57) | 0 | 0 |
| Arthralgia | 2 | 0 | 0 | 2 | 0 | 0 | 2 (29) | 0 | 0 |
| Fatigue | 1 | 1 | 0 | 3 | 0 | 0 | 2 (29) | 1 (14) | 0 |
| Feverishness | 2 | 0 | 1^*^ | 2 | 0 | 0 | 2 (29) | 0 | 1 (14) |
| Headache | 1 | 0 | 1^*^ | 2 | 0 | 0 | 1 (14) | 0 | 1 (14) |
| Malaise | 3 | 0 | 0 | 3 | 0 | 0 | 4 (57) | 0 | 0 |
| Myalgia | 3 | 0 | 0 | 2 | 0 | 0 | 3 (43) | 0 | 0 |
| Nausea | 1 | 0 | 0 | 1 | 0 | 0 | 1 (14) | 0 | 0 |
| Pyrexia | 0 | 0 | 0 | 1 | 0 | 0 | 1 (14) | 0 | 0 |
| Other^†^ | 1 | 0 | 0 | 0 | 0 | 0 | 1 (14) | 0 | 0 |
