# Supplementary material for: Impact on Malaria Parasite Multiplication Rates in Infected Volunteers of the Protein-in-Adjuvant Vaccine AMA1-C1/Alhydrogel+CPG 7909
Source: PLoS One. 2011 Jul 22;6(7):e22271. doi: 10.1371/journal.pone.0022271 (PMC3142129; doi:10.1371/journal.pone.0022271)
Supplement: Protocol S1 — Trial Protocol. (PDF) [file pone.0022271.s004.pdf]

Study Reference: VAC 035

Title: A Phase I/IIa Study of the Safety, Immunogenicity and Parasite Growth Inhibitory Activity of AMA1-C1/Alhydrogel® + CPG 7909, an Asexual Blood Stage Vaccine for *Plasmodium falciparum* Malaria

Sponsor: University of Oxford

Chief Investigator: Professor Adrian V.S. Hill

Version 9.0

13th August 2010

Modification History

Version	Date	Author(s)
7	02/09/09	Susanne Sheehy, Alison Lawrie, Adrian Hill
7.1	13/10/09	Alison Lawrie
7.2	20/10/09	Susanne Sheehy
8	12/11/09	Susanne Sheehy, Christopher Duncan
8.1	08/02/10	Alison Lawrie
8.2	07/04/10	Christopher Duncan
9.0	13/08/10	Alison Lawrie

Modifications made since Version 8.2

Section	Title	Modification
Page 3<br><br>Synopsis<br>5<br><br><br>11.2<br><br>Glossary	Page 3<br><br>Synopsis<br>Selection and withdrawl of trial subjects<br>Number of Subjects<br><br>Glossary	The Malaria Vaccine Development Branch (MVDB) has been renamed as the Laboratory of Malaria Imunology and Vaccinology (LMIV)<br>Trial numbers reduced from 21 to 10<br><br>Reduction in trial length to 9 months<br><br>Numbers reduced to 7 vaccinees from 15 and 3 controls from 6.<br><br>This section was updated to 7 vaccinees and 3 controls<br>Addition of ORCRB to glossary

Title: A Phase I/IIa Study of the Safety, Immunogenicity and Parasite Growth Inhibitory Activity of AMA1-C1/Alhydrogel® + CPG 7909, an Asexual Blood Stage Vaccine for *Plasmodium falciparum* Malaria

Study Code: VAC035

EudraCT Number: 2007-005389-11

Chief Investigator:

Professor Adrian Hill
Centre for Clinical Vaccinology and Tropical Medicine (CCVTM)
Old Road
Oxford
OX3 7LJ
Tel: (01865) 857401
Fax: (01865) 857471
E-mail: adrian.hill@well.ox.ac.uk

Co-Investigator

Dr Ruth D. Ellis
Laboratory of Malaria Imunology and Vaccinology (LMIV)
National Institute for Allergy and Infectious Diseases/ National Institutes for Heath (NIAID/NIH)
Rockville, Maryland, USA
Tel 301-435-3064
E-mail: ellisru@niaid.nih.gov

Principle Investigator

Dr Saul N. Faust
Wellcome Trust Clinical Research Facility
University of Southampton
C Level, West Wing, Mailpoint 218
Southampton University Hospitals NHS Trust
Tremona Road
Southampton
SO16 6YD
Tel: 023 8079 6883
Fax: 023 8079 5023
Email: s.faust@soton.ac.uk

Trial Centres:

Centre for Clinical Vaccinology and Tropical Medicine (CCVTM)
University of Oxford
Churchill Hospital
Old Road
Headington
Oxford
OX3 7LJ
Tel: (01865) 857401

Wellcome CRF, Southampton
Southampton University Hospitals NHS Trust
Southampton
SO16 6YD
Tel:023 8079 4989

Sponsoring Institution:

University of Oxford
University Offices
Wellington Square
Oxford
OX1 2JD

Project Managers:

Dr Alison Lawrie
University of Oxford
CCVTM, Oxford.Tel: 01865 857382
Email: Alison.lawrie@ndm.ox.ac.uk

Dr Yimin Wu
Malaria Vaccine Development Branch (MVDB)
Rockville, Maryland, USA
Tel +1-301-435 2654
Yiwu@niaid.nih.gov

External monitors:

Ceri McKenna and Carole Hall
Appledown Clinical Research Ltd.
Orchard's End,
Greenland's Lane,
Prestwood,
Gt. Missenden,
Bucks.
HP16 9QX
Tel/Fax: +44 (0)1494 867676

Local Safety Committee Chair:

Dr. Brian Angus
Nuffield Department of Clinical Medicine
University of Oxford
John Radcliffe Hospital
Oxford
OX3 9DU.
Tel: (01865) 225219
Fax: (01865) 225571
e-mail: brian.angus@ndm.ox.ac.uk

Investigator Agreement

"I have read this protocol and agree to abide by all provisions set forth therein.

I agree to comply with the International Conference on Harmonisation Tripartite Guideline on Good Clinical Practice."

Principal Investigator<br>PROF. ADRIAN HILL	Investigator Signature	Date

Confidentiality Statement

This document contains confidential information that must not be disclosed to anyone other than the Sponsor, the Investigator Team, and members of the Independent Ethics Committee. This information cannot be used for any purpose other than the evaluation or conduct of the clinical investigation without the prior written consent of Adrian Hill.

TABLE OF CONTENTS

Study Reference: VAC 035 1
Sponsor: University of Oxford 1
Chief Investigator: Professor Adrian V.S. Hill 1
1. SYNOPSIS 9
2. GLOSSARY 11
3. BACKGROUND AND RATIONALE 13
3.1 Malaria Vaccines 13
3.2 Previous work 13
4. OBJECTIVES 18
4.1 Primary Objective 18
4.2 Secondary Objectives 18
4.3 Tertiary Objectives 18
5. SELECTION AND WITHDRAWAL OF TRIAL SUBJECTS 19
5.1 Inclusion Criteria 19
5.2 Exclusion Criteria 19
5.3 Withdrawal of Patients/Subjects 20
6. TRIAL DESIGN 21
6.1 Details of Study Design and Procedures 21
6.2 Minimisation of Bias 27
6.3 Study Vaccine 27
6.4 Duration of Study 28
6.5 Definition of End of Trial 28
6.6 Discontinuation Criteria 29
6.7 Accountability of the Study Treatment 29
6.8 Randomisation 30
6.9 Source Data 30
7. TREATMENT OF TRIAL SUBJECTS 31
7.1 Name and description of investigational products 31
7.2 Known and potential risks and benefits 31
7.3 Precautions to minimise risk 32
7.4 Dosage and route of administration 32
7.5 Concomitant Medication 33

© The University of Oxford 2010

7.6 Compliance with Dosing Regime 33

8. MALARIA CHALLENGE 34
8.1 Background 34
8.2 Known and potential risks 34
8.3 Precautions to minimise risk from malaria 35
8.4 Previous experimental use of malaria-infected red blood cells 36
8.5 Storage conditions 36
8.6 Administration of the Inoculum 36

9. OUTCOME MEASURES 37
9.1 Levels of Parasite Growth Inhibitory Assay (GIA) 37
9.2 Parasite Multiplication Rates 37
9.3 Safety data 37
9.4 Immunological Assays 37
9.5 Parasite Identification by Thick Film Analysis 37

10. ASSESSMENT OF SAFETY 38
10.1 Safety Parameters 38
10.2 Methods and Timing of Safety Assessments 38
10.3 Definitions 38
10.4 Serious Adverse Event Reporting Procedures 40
10.5 Reporting Procedures for All Adverse Events 41
10.6 Local Safety Committee (LSC) 41
10.7 Stopping Criteria 41

11. STATISTICS 43
11.1 Description of Statistical Methods 43
11.2 The Number of Subjects 43
11.3 The Level of Statistical Significance 43

12. DIRECT ACCESS TO SOURCE DATA/DOCUMENTS 44
13. QUALITY CONTROL AND QUALITY ASSURANCE PROCEDURES 45
14. ETHICS 46
14.1 Declaration of Helsinki 46
14.2 ICH Guidelines for Good Clinical Practice 46
14.3 Informed Consent 46

14.4 Independent Ethics Committees 46

14.5 Patient/subject Confidentiality 46

15. DATA HANDLING AND RECORD KEEPING 47

15.1 Data Handling 47

15.2 Record Keeping 47

15.3 Source Data and Case Report Forms (CRFs) 47

15.4 Data Protection 47

15.5 Use, Storage, and Tracking of Specimens and Data 47

16. FINANCING AND INSURANCE 49

16.1 Compensation / Expenses 49

16.2 Insurance 49

17. PUBLICATION POLICY 50

APPENDIX A: SCHEDULE OF ATTENDANCES 51

APPENDIX B: LABORATORY VALUES FOR EXCLUSION 52

APPENDIX C: GRADING SCALES FOR ADVERSE EVENTS 53

Appendix D: Toxicity Table for Grading Laboratory Adverse Events 54

Appendix E: Table for Grading Adverse Events 55

1. SYNOPSIS

**Trial Title**	"A Phase I/IIa Study of the Safety, Immunogenicity and Parasite Growth Inhibitory Activity of AMA1-C1/Alhydrogel® + CPG 7909, an Asexual Blood Stage Vaccine for *Plasmodium falciparum* Malaria"
**Trial Centres**	Centre for Clinical Vaccinology and Tropical Medicine (CCVTM)<br>Old Road, Headington, Oxford, OX3 7LJ, UK<br>Wellcome CRF, Southampton University Hospitals NHS Trust, Southampton<br>SO16 6YD
**Trial Identifier**	VAC035
**Clinical Phase**	I / IIa
**Finished Product**	AMA1-C1/Alhydrogel® + CPG 7909
**Active Ingredients**	AMA1-C1: A mixture of two recombinant synthetic AMA1 proteins from *Plasmodium falciparum* strains 3D7 and FVO<br>Alhydrogel®: An aluminium-based adjuvant, on which the two AMA1 proteins are formulated<br>CPG 7909: An oligodeoxynucelotide, which enhances B cell responses to co-administered antigens and induces a Th1 biased response
**Trial Design**	Controlled clinical trial<br>Vaccination followed by blood stage malaria challenge<br>With the option of conducting the study in two parts if volunteers' availability makes this preferable.
**Trial Population**	Healthy, malaria naïve males and non-pregnant females aged 18 - 50
**Planned Sample Size**	Group 1: 7 subjects – 2 doses of AMA1-C1/Alhydrogel® + CPG 7909 at 0 and 8 weeks<br>Group 2: 3 control subjects for malaria challenge – these volunteers will not receive any vaccinations, but will confirm the infective efficacy of the challenge system.<br>Total: 10volunteers (All groups undergo malaria challenge)
**Follow-up duration**	Group 1: 238 days<br>Group 2: 168 days (challenge phase alone)<br>Note: follow up duration is an estimate and may vary in accordance with the specified time windows for each attendance.
**Blood Sampling Schedule**	Group 1: PS, S, D0, D7, D14, D28, D56, D63, D70, D71, twice daily from D72 – D82, once daily from D83 – D86, D98, D238.<br>Total of 633mL<br>Group 2: PS, S, D56, D63, D70, D71, twice daily from D72 – D82, once daily from D83 – D86, D98, D238.<br>Total of 359 mL
**Planned Trial Period**	9months
**Primary Objective**	To demonstrate a correlation between *in vitro* growth inhibition assay and parasite multiplication rate *in vivo*
**Secondary Objective**	To detect differences in the parasite multiplication rate responses between unvaccinated control subjects and volunteers vaccinated with AMA1-C1/Alhydrogel® + CPG 7909

**Tertiary Objectives**	To assess the safety and reactogenicity of the AMA1-C1/Alhydrogel® + CPG 7909 vaccine<br>To assess immunogenicity in response to vaccination
**Investigational Product**	AMA1-C1/Alhydrogel® + CPG 7909
**Form**	Liquid suspension
**Dose**	AMA1-C1/Alhydrogel® + CPG 7909 = 0.55 mL<br>(AMA1-C1 = 80 μg; Alhydrogel 800 μg, CPG 7909 = 564 μg)<br>(from 0.7 mL AMA1-C1/Alhydrogel® mixed with 0.08 mL CPG 7909)
**Route**	Intramuscular injection in the deltoid region of the upper arm
**Lot Number**	AMA1-C1/Alhydrogel® Lot BDP L0803002; CPG 7909 Lot BDP L08080002

2. GLOSSARY

**ADR**	Adverse drug reaction
**AMA1**	Apical Membrane Antigen 1
**ANA**	Antinuclear antibody
**AVA**	Anthrax vaccine adsorbed
**3D7**	Clone of NF54 strain of *Plasmodium falciparum*
**AE**	Adverse event
**ALT**	Alanine aminotransferase
**ALP**	Alkaline phosphatase
**β-HCG**	beta - human chorionic gonadotrophin
**BPD**	Biopharmaceutical Development Program
**CCVTM**	Clinical Centre for Vaccinology and Tropical Medicine
**cGMP**	Current Good Manufacturing Practice
**CI**	Chief Investigator
**CMV**	Cytomegalovirus
**CPG**	Cytosine-Guanine repeat sites
**CRF**	Case report form
**dsDNA**	double-stranded DNA
**EBV**	Epstein Barr Virus
**ELISA**	Enzyme-linked immunosorbent assay
**ELISPOT**	Enzyme-linked immunospot
**FBC**	Full blood count
**FDA**	US Food and Drug Administration
**FVO**	Clone of *Plasmodium falciparum*
**GCP**	Good Clinical Practice
**GIA**	Growth Inhibitory Activity
**GP**	General Practitioner (Family Doctor)
**IEC**	Independent Ethics Committee
**IFN-γ**	Gamma interferon
**IM**	intramuscular
**IND**	Investigational New Drug application
**IRB**	Institutional Review Board
**HBsAg**	Hepatitis B surface antigen
**HBV**	Hepatitis B virus
**HCV**	Hepatitis C virus
**HIV**	Human immunodeficiency virus
**HLA**	Human leukocyte antigen
**LFT**	Liver function test
**LSC**	Local safety committee
**MHRA**	Medicines and Healthcare products Regulatory Agency
**MSP**	Merozoite Surface Protein
**MVA**	Modified vaccinia Virus Ankara
**MVDB**	Malaria Vaccine Development Branch
**NIAID**	National Institute of Allergy and Infectious Diseases
**NIH**	National Institutes of Health
**NHS**	National Health Service
**NYVAC**	Highly attenuated vaccinia virus
**ODN**	Oligodeoxynucleotide
**ORCRB**	**Old Road Campus Research Building**
**OxREC**	Oxfordshire Research Ethics Committee
**PCR**	Polymerase chain reaction
**RF**	Rheumatoid Factor
**SOP**	Standard Operating Procedure
**SAE**	Serious adverse event
**SAIC**	Science Applications International Corporation
**SUSAR**	Serious Unexpected Adverse Reaction
**TLR9**	Toll-like Receptor 9

**WBC**	White blood cell
**WRAIR**	Walter Reed Army Institute of Research

© The University of Oxford 2010

3. BACKGROUND AND RATIONALE

3.1 Malaria Vaccines

As reported by the World Health Organization in 2002, the worldwide incidence of malaria is approximately 300 million clinical cases annually, with approximately one million deaths per year attributed to malaria alone or in combination with other diseases. (WHO, 2002) Most of the mortality occurs among children under 5 years of age in sub Saharan Africa. Of the four species of malaria that infect humans, *Plasmodium falciparum* is responsible for the majority of these deaths. Mounting drug resistance of the malaria parasite, as well as widespread resistance of mosquitoes to insecticides make these control strategies increasingly unrealistic. A vaccine that would reduce both mortality and morbidity secondary to *P. falciparum* infection would be a valuable resource in the fight against this disease.

It has been possible with suitable adjuvantation to induce protective antibody responses with this recombinant antigen in several simian and murine studies. (Anders et al., 1998; Narum et al, 2000; Stowers et al., 2002). Mouse antibodies induced by an AMA1 peptide show growth inhibitory activity (Mueller et al., 2003). Several human clinical trials have been carried out with recombinant AMA1 and various adjuvants, and have generated antibodies with varying levels of activity in growth inhibition assays (see section 3.2.5).

Viral-vectored vaccines for AMA1 have been shown to be immunogenic in mouse models(Mueller et al., 2003; Miao, 2006). In the macaque based *P. knowlesi* model the US Navy group have shown significant efficacy using a combination of DNA and highly attenuated vaccinia virus (NYVAC) vectored vaccines. This has been attributed to the use of AMA1 with MSP-1_{42}. (Rogers et al., 2002). Similar pre clinical work using the blood stage antigen MSP-1 as an insert in an adenovirus prime – modified vaccinia virus Ankara (MVA) boost regime has shown the ability to induce high titre protective antibody responses in the murine *P. yoelii* model. In addition, using this vaccination regime we can demonstrate multi-stage immunity, with MSP-1_{42} specific T cells providing clearance of late liver stage parasites (Draper et al., 2007). AMA1 should also provide such multi stage immunity, given its expression by the sporozoite (Silvie et al., 2004) also presumably by merozoites during development of late liver stage parasites.

AMA1 polymorphism presents a potential issue for the development of a widely effective vaccine. Most allelic diversity is reported in domains I and II in response to immune selection pressure (Polley and Conway, 2001). Most studies of naturally exposed individuals have shown both strain-specific and cross-reactive antibody responses, but only responses against the entire ectodomain, rather than individual epitopes correlate with protection(Polley et al., 2004)

CPG 7909 is a short synthetic oligodeoxynucelotide with all nucleotides linked with phosphorothioate bonds. Recently, it has been shown that polynucleotides containing this motif can significantly enhance B cell responses to co-administered antigens via interactions with toll-like receptor 9 (TLR9). Additionally, CPG 7909 is known to induce a Th1-biased immune response which has been shown to be more effective for inducing cell-mediated immune responses. CPG 7909 has previously been administered to humans on its own as an investigational cancer therapeutic agent and as a vaccine adjuvant with two licensed vaccines to healthy adult subjects without significant safety concerns (see Section 3.2.3). Results in humans show that the combination of AMA1-C1/Alhydrogel® + CPG 7909 boosts antibody levels significantly relative to AMA1-C1/Alhydrogel® alone, with correspondingly enhanced parasite growth inhibition. This study aims to determine whether this improved parasite growth inhibition *in vitro* correlates with reduced parasite multiplication rates *in vivo*.

3.2 Previous work

3.2.1 Preclinical studies with AMA1-C1/Alhydrogel®

A total of 3 individual preclinical trials have been conducted to assess the safety and immunogenicity of the AMA1-C1/Alhydrogel® + CPG 7909 vaccine prior to human clinical trials. This combination has been tested by IM administration in mice, rats, guinea pigs, and

rabbits. The AMA1-C1/Alhydrogel® + CPG 7909 formulation was more immunogenic in all species other than rabbits as compared to AMA1-C1/Alhydrogel® alone. No clinically significant adverse effects have been observed.

3.2.2 Clinical trials with AMA1-C1/Alhydrogel®

There have been four human clinical trials to date using the recombinant AMA1-C1/Alhydrogel® vaccine without CPG 7909.

The first Phase 1 clinical trial to use AMA1-C1/Alhydrogel® was performed at Johns Hopkins University (Baltimore, MD)(Malkin et al., 2005). Thirty subjects received up to 3 immunisations (Day 0, 28 and 180) with either 5, 20, or 80 µg doses of AMA1-C1/Alhydrogel® in an open label dose escalating study. No safety concerns were identified. Adverse events related to vaccination were limited to local injection site reactions, which were mild to moderate in severity. There was a dose response with the 80 µg group giving the highest antibody levels at day 42 (14 days after the second vaccination) and at day 194 (14 days after the third vaccination). A low level of growth inhibition, correlating with antibody titer, was demonstrated in vaccine recipients after three doses.

The second clinical trial using AMA1-C1/Alhydrogel® was started in Mali in May 2004. This Phase 1 study was a randomized, double blind, controlled trial conducted by the University of Bamako. Fifty-four healthy Malian adults were randomized 2:1 to receive either AMA1-C1/Alhydrogel® at either 5, 20, or 80 µg doses, or the Recombivax® Hepatitis B vaccine. Study participants received 3 immunisations on study days 0, 28, and 360. No vaccine-related serious or Grade 3 events were observed. Antibody responses through Study Day 270 showed a dose response, with higher titers after the second vaccination in the group receiving 80 µg AMA1-C1/Alhydrogel compared to the lower dose groups. Antibody levels peaked 14 days following the second vaccination, and subsequently decreased, reaching baseline levels one year later at the time of the third vaccination. Unlike after the second vaccination, the third vaccination at Day 360 induced little or no increase in antibody levels.

The third trial was conducted at the NIH Clinical Center (Rockville, MD). This study planned to vaccinate 12 healthy adults with 80 µg of AMA1-C1/Alhydrogel® and 6 with Alhydrogel® alone. Enrolment was closed at 6 volunteers due to difficulty with recruitment; 4 volunteers received AMA1-C1/Alhydrogel® and 2 received Alhydrogel®. The objectives of this study were to observe B cell responses to vaccination. No safety concerns were identified with the 6 volunteers vaccinated.

The fourth trial was a Phase 1/2 randomized, controlled study of the safety and immunogenicity of AMA1-C1/Alhydrogel® in Malian children conducted at the University of Bamako. In Phase 1, 36 children (Cohort 1) were randomized to receive either 20 µg AMA1-C1/Alhydrogel®, 80 µg AMA1-C1/Alhydrogel®, or the comparator vaccine (Hiberix™), with two doses given one month apart. Phase 2 enrolled an additional 300 children, separated into Cohort 2 (60 children) and Cohort 3 (240 children). These children were randomized to receive either 80 µg AMA1-C1/Alhydrogel® or the comparator and were followed through a malaria transmission season to assess biological impact of the vaccine, as measured by a reduction in parasitemia. No impact on frequency of parasitemia >3000 or on density of parasites was seen in the first transmission season (to study day 154). A statistically significant decrease in hemoglobin during clinical malaria episodes and an increase in anemia (hemoglobin < 8.5 g/dL) were seen in the group who received AMA1-C1/Alhydrogel, although these were not significant after correction was made for multiple tests. The impact on hemoglobin and anemia persisted when data to day 365 were analyzed. The original protocol planned to follow participants for 12 months after first vaccination. After the close of the study 4 children had events which would have been considered serious adverse events, with two deaths. All were in the group which received AMA1-C1/Alhydrogel (refer to Investigator Brochure for further details). The protocol was subsequently reopened and additional safety data for the following transmission season was obtained. No significant differences in hemoglobin, frequency of anemia, or severity of malaria were detected in the extended follow up period.

3.2.3 Previous clinical experience with CPG 7909 (VaxImmune®):

This CPG 7909 motif has been administered to humans in combination with Engerix-B® Hepatitis B vaccine, Fluarix® killed influenza vaccine, and anthrax vaccine adsorbed (BioThrax®) . Results from these clinical trials indicate that the addition of CPG 7909 is safe, and in the case of Engerix-B® and BioThrax®, induced significantly earlier and stronger antibody responses than the vaccine alone.

The Engerix-B® trial in healthy adults was a randomized, double-blind study in 56 subjects in Canada. . The most frequently reported adverse events were injection site reactions (pain and erythema), flu-like symptoms and headache. All adverse events were mild or moderate in severity. One volunteer experienced a hypersensitivity-type reaction immediately following the third dose of Engerix-B® + 1.0 mg of CPG 7909. The symptoms included warmth, weakness, nausea, and dizziness. The symptoms were transient and resolved without treatment. One volunteer who received Engerix-B® + 1.0 mg of CPG 7909 had moderate elevations in anti-dsDNA which was initially detected two weeks after the second and third vaccinations. The anti-dsDNA returned to normal prior to receipt of the third dose and was normal at the end of the study. The volunteer was asymptomatic, and ANA and rheumatoid factor remained negative throughout.

Engerix-B® mixed with CPG 7909 was also evaluated in a randomized, double-blind controlled trial in 58 HIV infected adults in Canada. The most common adverse events were local injection site reactions and influenza-like symptoms. Two serious adverse events were reported in individuals who received Engerix-B® with CPG 7909: one who developed unstable angina and myocardial infarction 2 and 4 months after the last vaccination respectively, and one who had laparoscopy for chronic pelvic inflammatory disease 16 days following vaccination. Laboratory abnormalities included transient declines in total lymphocytes and CD4 cells. Two subjects receiving Engerix-B® plus CPG 7909 and two subjects receiving CPG 7909 alone had transient elevations of anti-dsDNA; there were no clinical findings of autoimmunity.

The Fluarix® trial was a randomized, controlled, double-blind study in 60 healthy subjects in Canada . Subjects received either the licensed Fluarix® vaccine with or without CPG 7909, or 1/10th the dose of Fluarix® with or without CPG 7909. The most frequently reported adverse events were injection site pain, headache, myalgia and fatigue. All injection site reactions were mild or moderate in severity, with the exception of one volunteer in the 1/10th dose Fluarix® + CPG 7909 group who reported severe pain which resolved without treatment within four days. Transient reductions in total WBC, neutrophils, lymphocytes, eosinophils and platelets were observed in all four study arms 2 days post-vaccination, with larger reductions in total white blood cells (WBC) in the CPG 7909 groups. None of these results were felt to be clinically significant.

CPG 7909 combined with AVA, the licensed anthrax vaccine adsorbed (BioThrax®), was evaluated in a randomized, controlled, double-blind study in 69 healthy adults. Local injection site and systemic symptoms were expected and were the most common adverse events. There was a trend to greater frequency and severity of adverse events in the AVA plus CPG 7909 group compared to the AVA alone and CPG 7909 alone groups but this was not statistically significant. Grade 1 leukopenia and hypokalaemia were seen in all study arms.

A Phase 1 clinical trial of the blood stage malaria vaccine recombinant $MSP1_{42}$-C1/Alhydrogel® was conducted at the Johns Hopkins Center for Immunization (Washington, DC). Sixty subjects were randomized to receive three IM injections a month apart of a low (40 µg) or high (80 µg) dose of $MSP1_{42}$-C1/Alhydrogel® with and without CPG 7909. All local adverse events were mild or moderate with the exception of severe erythema in one volunteer in the high dose $MSP1_{42}$-C1/Alhydrogel®+CPG 7909 group. All solicited adverse events were either mild or moderate and no serious adverse events were reported. Preliminary analysis suggests that adverse events such as malaise, myalgia, and arthralgia were more common in the groups receiving CPG 7909. Transient neutropenia developed in 9 volunteers receiving the vaccine with CPG 7909 (some volunteers had neutropenia after more than one vaccination); in only two individuals was the neutropenia graded as moderate. Neutropenia was also observed in one volunteer that received the vaccine alone.

Neutropenia resolved by day 7 post-vaccination for all volunteers. Autoantibody studies (ANA, anti-dsDNA, and RF) were measured at screening and at baseline. Anti-dsDNA was also measured at each day of vaccination, 7 days after each vaccination, and at Study Days 84 and 236 (a total of 8 times excluding screening); ANA and RF were checked on those days only if anti-dsDNA was positive. Two (2) volunteers had elevated ANA (>1:80) at baseline and were not revaccinated; 28 volunteers had low ANAs (1:40) at baseline and were continued in the study without the subsequent development of safety issues. No positive anti-dsDNA results occurred.

3.2.4 Previous clinical experience with AMA1-C1/Alhydrogel® + CPG 7909:

A Phase 1 clinical trial of recombinant AMA1-C1/Alhydrogel®+CPG 7909 was conducted at the University of Rochester (Rochester, NY). In this trial 75 healthy, malaria-unexposed adults received up to 3 immunizations of either 20 µg of AMA1-C1/Alhydrogel® + 500 µg CPG 7909 (N = 15), 80 µg of AMA1-C1/Alhydrogel® + 500 µg CPG 7909 (N = 30), or 80 µg of AMA1-C1/Alhydrogel® (N = 30), at 0, 4 and 8 weeks. Most reported local and solicited adverse events were graded as either mild or moderate in severity. No serious adverse events occurred that were definitely, probably, or possibly related to vaccination. Local reactions were all mild or moderate with the exception of one volunteer in the 80 µg AMA1-C1/Alhydrogel® group, who reported 120 mm induration at the injection site in a symptom diary. This report was not confirmed by objective findings in the study clinic. Preliminary analysis suggests that solicited systemic events such as fatigue and malaise were more common in the CPG 7909 groups, and were more likely to be moderate or severe in the higher dose (80 µg) AMA1-C1/Alhydrogel®+CPG 7909 group than the lower dose. Local and solicited systemic adverse events were not more frequent with subsequent doses.

Several volunteers (mostly female) had drops in hemoglobin, thought to be related to menstruation and the volume of blood drawn for the study. Autoantibodies (ANA, anti-dsDNA, and RF) were checked at screening, at each day of vaccination, at Day 7 after each vaccination, at 28 days after the last vaccination, and at the end of the study. Three volunteers were withdrawn due to positive ANA; two of these were positive (1:80) at baseline and one was positive at the day of second vaccination (1:1280); all returned to normal with some low fluctuations. (The protocol was amended after the enrollment of Cohort 2 to allow continued participation if ANA was positive, as long as other autoimmune laboratory values were normal.) One volunteer was withdrawn due to positive anti-dsDNA (82 IU), which subsequently returned to normal. No volunteers had clinical evidence of autoimmune disease.

Preliminary immunogenicity results suggest 8 to 10 fold enhancement of the antibody response in volunteers receiving CPG 7909. When tested for functional activity by *in vitro* inhibition of parasite invasion, IgG isolated from serum of volunteers receiving AMA1-C1/Alhydrogel® + CPG 7909 was significantly more effective against both FVO and 3D7 parasites than an equal concentration of IgG from volunteers receiving vaccines adjuvanted with Alhydrogel® alone. Growth inhibition of >90% was demonstrated in some volunteers' sera.

The studies described above have all used CPG 7909 in a phosphate buffer. Studies at MVDB indicate that the binding of the AMA1-C1 protein to Alhydrogel® is optimized when the CPG 7909 is formulated in a saline buffer. A Phase 1 study of AMA1-C1 with CPG 7909 in a saline buffer was conducted by the Center for Immunization Research to evaluate safety and immunogenicity of the saline formulation, and to demonstrate that the addition of CPG 7909 in saline enhanced the immune response to AMA1-C1 in a manner similar to that seen with the addition of CPG 7909 in phosphate. Twenty-four (24) volunteers were enrolled and received two vaccinations of either AMA1-C1/Alhydrogel® +CPG 7909(phosphate) or AMA1-C1/Alhydrogel® +CPG 7909(saline), on a 0,1 or 0,2 month schedule in a 2x2 design. No safety issues were identified. Responses were similar in the groups receiving the phosphate and saline formulations, and were higher in the groups receiving vaccinations on a two month rather than one month schedule.

A Phase 1 study in malaria-exposed adults in Mali completed vaccinations in December, 2007. Twenty-four (24) volunteers were enrolled and randomized 1:1 to receive either AMA1-C1/Alhydrogel® or AMA1-C1/Alhydrogel ® + 500 µg CPG 7909 (saline). Two vaccinations were given one month apart. One volunteer was withdrawn from vaccinations due to a Grade 1 hypersensitivity reaction; this volunteer had a previously undisclosed history of allergic reactions requiring treatment with steroids. No other safety issues have been identified thus far. Preliminary immunogenicity results suggest a 2.5 fold increase in antibody responses in volunteers receiving CPG 7909.

The current study will use CPG 7909 in a saline buffer.

3.2.5 Previous clinical experience with Blood stage Challenge

Initial development of the blood inoculum to be used in this study and its use in five volunteers is described by Cheng et al (Cheng et al, 1997). (Development of the inoculum is summarized in Protocol Section 8.1.1.) In that study, two volunteers infected with the inoculum were treated based on positive PCR prior to development of symptoms; the other three were treated when symptomatic. All recovered fully with rapid decline of parasitemia after treatment with chloroquine. The inoculum was also administered to 17 volunteers to test a mixture of three recombinant *Plasmodium falciparum* blood stage antigens for ability to reduce the initial growth rate of parasites. All volunteers developed parasitemia detected by PCR and all were treated before the development of symptoms. The inoculum was used in a third study to determine whether or not development of cell-mediated immunity to blood stage infection could be induced by exposure to malaria parasites. Four volunteers were repeatedly infected and treated with atovaquone/proguanil prior to the development of symptoms, and then received another inoculation as the test challenge, subsequent to which they were treated with chloroquine. One volunteer was removed from the study due to the development of a slightly low neutrophil count. No other unexpected adverse events were reported in any of these studies.

Oxford University has previously undertaken a malaria challenge of five healthy human volunteers using this inoculum. This study was approved in 2003 by the Oxfordshire Research Ethics Committee (ref. C03.061) As expected, the results showed less variation in rate of rise of parasitaemia than is typically experienced with a liver-stage (or sporozoite) malaria challenge (personal communication, F. Sanderson). The trial was successful, with all volunteers being diagnosed with malaria between the morning of day 7 and the morning of day 9 after inoculation. The symptoms of malaria experienced by volunteers at and around the point of diagnosis were as expected. No serious adverse events (SAEs) were reported. All volunteers were clinically well after antimalarial treatment when reviewed in clinic at day 42 after inoculation. During the trial one volunteer became mildly anaemic but this was transient, with a normal haemoglobin on repeat testing one week later; and one volunteer had a raised bilirubin of 35 µmol/L on two occasions. All volunteers successfully completed follow-up.

In this study volunteers will be treated when parasites are detected by microscopy, or when symptomatic, or at Day 16 (Section 5.1.1), rather than when parasites are first detected by PCR. This will maximize the number of data points available to calculate in vivo parasite multiplication rates.

4. OBJECTIVES

4.1 Primary Objective

To demonstrate a correlation between *in vitro* growth inhibition assay and parasite multiplication rate *in vivo*

4.2 Secondary Objectives

To detect differences in the multiplication rate responses between unvaccinated control subjects and volunteers vaccinated with AMA1-C1/Alhydrogel® + CPG 7909

4.3 Tertiary Objectives

I. To assess the safety and reactogenicity of the AMA1-C1/Alhydrogel® + CPG 7909 vaccine

II. To assess immunogenicity in response to vaccination

5. SELECTION AND WITHDRAWAL OF TRIAL SUBJECTS

The study will be conducted in healthy adults ages 18 to 50, with no history of malaria or recent travel to malarious areas. Two centres will participate in recruitment and vaccination (Oxford CCVTM and Southampton CTU). Recruitment will proceed at each centre until the total number of volunteers required is reached (7 vaccinees, 3 controls). Volunteers who have previously registered an interest in participating in clinical trials in Oxford or Southampton and who have agreed to be contacted regarding future clinical trials will be invited to pre-screening.

5.1 Inclusion Criteria

The patient/subject must satisfy all the following criteria to be eligible for the study:

- Subject is willing and able to give informed consent for participation in the study
- Healthy, non pregnant adult aged 18 - 50 years
- Resident in or near Oxford for the duration of the challenge study
- Seropositive for CMV and EBV
- Female subjects of child bearing potential must be willing to ensure that they practice effective contraception during the study
- Males must be willing to use barrier contraception from day of first vaccination onwards until 3 months after the second vaccination
- Able (in the Investigator's opinion) and willing to comply with all study requirements
- Willing to allow his or her General Practitioner and consultant, if appropriate, to be notified of participation in the study
- Agreement to permanently refrain from blood donation.

5.2 Exclusion Criteria

The patient/subject may not enter the study if ANY of the following apply:

- Any clinically significant deviation from the normal range in biochemistry or haematology blood tests or in urine analysis as defined in Appendix B
- Female patient/subject who is pregnant, lactating or planning pregnancy during the course of the study
- Healthy volunteers who have participated in another research study involving an investigational product in the past 12 weeks
- Subjects who have previously received an investigational malaria vaccine
- History of malaria chemoprophylaxis with chloroquine within 5 months prior to the planned challenge, with Lariam within 6 weeks prior to the challenge, and Riamet® within 2 weeks prior to the challenge
- Travel to a malaria endemic area within the previous 6 months
- Planned travel to malarious areas during the study period
- Any history of malaria

- Contraindication to both anti-malarial drugs (Riamet® and chloroquine)
    - concomitant use of other drugs known to cause QT-interval prolongation, (e.g. macrolides, quinolones, amiodarone etc)
- An estimated ten year risk of fatal cardiovascular disease of ≥5%, as estimated by the Systematic Coronary Risk Evaluation (SCORE) system (Conroy 2003)
- Family history of sudden cardiac death
- History of cardiac arrhythmia or prolonged QT syndrome
- Any history of severe allergic reaction or anaphylaxis
- History of a known allergy to nickel
- Any confirmed or suspected immunosuppressive or immunodeficient state, including HIV infection; asplenia; recurrent severe infections and chronic (more than 14 days) immunosuppressant medication within the past 6 months
- History or evidence of pre-existing autoimmune or antibody mediated disease or laboratory evidence of possible autoimmune disease (defined as anti-dsDNA ≥ 25 IU/mL)
- Seropositive for hepatitis B surface antigen (HBsAg) or antibodies to hepatitis C virus
- Any on-going chronic illness requiring hospital specialist supervision
- Administration of immunoglobulins and/or any blood products within the three months preceding the planned administration of the vaccine candidate
- History of or current intravenous drug abuse
- Suspected or known current alcohol abuse as defined by an alcohol intake of greater than 42 units every week
- Any other significant disease or disorder which, in the opinion of the Investigator, may either put the subject at risk because of participation in the study, or may influence the result of the study, or the subject's ability to participate in the study.
- Investigator assessment of lack of willingness to participate and comply with all requirements of the protocol

5.3 Withdrawal of Patients/Subjects

Subjects may withdraw or be withdrawn for any of the reasons given in the Discontinuation Criteria (Section 6.6). The reason for withdrawal will be recorded in the CRF. If the subject is withdrawn due to an adverse event, the Investigator will arrange for follow-up visits or telephone calls until the adverse event has resolved or stabilised.

Any subject who is withdrawn from the study may be replaced, if that is possible within the specified time frame.

Volunteers who withdraw after the point of challenge must take a full course of antimalarial medication and attend for two further visits for clinical review and blood sampling to ensure all parasites are killed.

The Local Safety Committee (LSC) may recommend withdrawal of subjects (see Section 10.6).

6. TRIAL DESIGN

6.1 Details of Study Design and Procedures

The study is an open Phase I/IIa clinical trial in healthy, malaria-naïve adult subjects designed to examine the relationship between malaria parasite growth rates following infection with an *in vitro* assay of parasite growth inhibition.

The novel malaria vaccine AMA1-C1 formulated on Alhydrogel® co-administered with CPG 7909 will be used to induce *P. falciparum*-specific antibodies. Twenty-one subjects will be enrolled, of which fifteen (group 1) will receive two vaccinations two months apart, and six (group 2) will act as unvaccinated control volunteers. Two weeks after administration of the second vaccine, both groups will be infected with blood stage malaria parasites under controlled conditions. Malaria eradication treatment will be given within the next 16 days.

There is the option of conducting the study in two parts if volunteers availability makes this preferable.

6.1.1 Challenge endpoints

Following malaria challenge, volunteers will be treated with a course of antimalarial medication as soon as the primary or secondary endpoint is reached.

The primary endpoint is:

- One thick smear positive for one or more parasites during the 16 day observation period.

The secondary endpoint is:

- Subject is manifesting typical clinical symptoms or signs of malaria in the opinion of the Principal Investigator (such as fever >37.5°C, rigors, moderate or severe myalgia, in the absence of another obvious cause) and has negative blood smears but has a PCR result that is positive for malaria.

If, on the morning of day 16 neither endpoint has been reached, the volunteer will be declared protected from malaria.

6.1.2 Informed Consent

All subjects will personally sign and date the informed consent form before any study specific procedures are performed. The information sheet will be made available to the volunteer at least 24 hours prior to the screening visit. At the screening visit the Investigator or clinical research nurse will fully inform the subject of all aspects of the trial, the potential risks and their obligations. The following general principles will be emphasised:

- Participation in the study is entirely voluntary
- Refusal to participate involves no penalty or loss of medical benefits
- The subject may withdraw from the study at any time
- The subject is free to ask questions at any time to allow him or her to understand the purpose of the study and the procedures involved
- The study involves research of an investigational vaccine and the novel adjuvant CPG 7909
- There is no direct benefit for participating
- The volunteer's GP will be contacted to corroborate their medical history

The aims of the study and all tests to be carried out will be explained by the Investigator or the experienced Clinical Research Nurse. The subject will be given the opportunity to ask about details of the trial, and will then have time to consider whether or not to participate. If they do

decide to participate, they will sign and personally date two copies of the consent form, one for them to take away and keep, and one for the Investigator. These forms will also be signed and dated by the Investigator or clinical research nurse.

6.1.3 Pre- Screening & Screening (Visit 1, ± 90 days, Visit 2 ± 90 days)

All volunteers will be subject to a two stage screening procedure. Consent will be taken to draw blood for EBV & CMV serology (5mls). If a stored serum sample for a volunteer is available and is less than 1 year old, this sample may be tested for CMV & EBV serology with the volunteer's consent, rather than taking a second blood sample. Volunteers seropositive for EBV & CMV will be invited to a screening visit. It is expected that approximately 80% of those pre-screened will be ineligible for the study due to lack of IgG specific to both CMV and EBV.

Volunteers seropositive for CMV & EBV will be invited to a screening visit, which may take place up to 90 days prior to vaccination. As part of the informed consent process before screening is undertaken, the screening process will be reviewed in detail with each prospective volunteer and all questions about the process answered.

A baseline medical history and physical examination will be performed. Inclusion and exclusion criteria will be checked. Vital signs will be checked. Subjects will be counselled by one of the Investigators for HIV, Hepatitis B and Hepatitis C testing. Subjects will have the opportunity to ask questions about the implications of HIV testing. Blood will be drawn for the following tests:

- Full blood count (including platelet count)
- Electrolytes, urea, creatinine, liver function tests, cholesterol
- HIV, hepatitis B, hepatitis C serology
- Anti-dsDNA;

Urinalysis will also be carried out to exclude glycosuria and significant proteinuria or haematuria, and a urinary pregnancy test (women only) will be performed. Females will be counselled to avoid pregnancy using a reliable method of contraception.

The subject's GP will be contacted with the written permission of the subject after satisfactory screening as notification that the subject is taking part in the study and to ascertain any significant medical history. At least two weeks will be allowed between screening and enrolment for the GP to reply.

All laboratory results will be reviewed and the reports signed by the Investigator who will record in the CRF whether they are normal, abnormal but not clinically significant, or abnormal AND clinically significant. In the latter case the eligibility of the subject will be reviewed.

6.1.4 Enrolment

The eligibility of the subject will be reviewed at the end of the screening visit and again when all results from the screening visit have been considered. If eligible, a Day 0 visit will be scheduled for the subject to receive the first dose of vaccine. Volunteers will not be considered enrolled in the study until they have received their first dose of vaccine, or until they have received the malaria challenge inoculum for the unvaccinated control volunteers. More volunteers may be invited to participate than are scheduled to be enrolled. If not enrolled, these alternates will be invited to return on the next day of enrolment. Alternates will be compensated for the study visit even if not enrolled, as described in Section 16.

6.1.5 Immunisation Procedure

Volunteers will receive two immunisations, on Days 0 and 56. The vaccine preparation procedure is described in section 6.3.4. After preparation of the injection site with alcohol, 0.55 mL of AMA1-C1/Alhydrogel® + CPG 7909 will be delivered by IM injection into the deltoid muscle. The second vaccination will be given in the opposite arm.

6.1.6 Day 0 (day of first vaccination)

The following procedures will be carried out on the day of vaccination:

- Verify that Informed Consent was obtained.
- Verify that all applicable eligibility criteria have been met.
- Perform abbreviated history.
- Obtain blood for haematology, biochemistry, anti-dsDNA, anti-AMA-1 antibody ELISA, Growth Inhibitory Activity (GIA), Human Leukocyte Antigen (HLA) testing and exploratory immunology (Section 9.4). An additional 5 mL of blood will also be obtained and serum will be stored. If subsequent to vaccination the volunteer develops clinical signs of autoimmune or other disease this sample will be used for baseline laboratory tests as indicated.
- Obtain a urine sample for to test for the presence of protein, glucose or blood.
- For females, perform a urinary β-hCG test. Ensure that test is negative before proceeding; a positive test will exclude the volunteer from the trial.
- Record vital signs (blood pressure, temperature and heart rate).
- Administer the vaccine.
- Observe for 30 minutes after vaccination to evaluate for immediate adverse reactions.
- Give subject oral thermometer, diary card and instructions for completion.

6.1.7 Day 3 ± 1

- Perform interim history and examine the injection site and any body systems felt to be necessary by the Investigator.
- Record vital signs.
- Review diary card.

6.1.8 Day 7 ± 2

- Perform interim history and examine the injection site and any body systems felt to be necessary by the Investigator.
- Record vital signs.
- Obtain blood for haematology, biochemistry, anti-AMA-1 antibody ELISA.
- Obtain a urine sample for to test for the presence of protein, glucose or blood.
- Collect Day 0-6 diary card.

6.1.9 Day 14 ± 3

- Perform interim history and examine the injection site and any body systems felt to be necessary by the Investigator.
- Record vital signs.

© The University of Oxford 2010

- Obtain blood for haematology, biochemistry, anti-AMA-1 antibody ELISA and exploratory immunology.
- Obtain a urine sample for to test for the presence of protein, glucose or blood, and for a urinary β-hCG test in females; a positive test will exclude the volunteer from receiving further vaccinations.

6.1.10 Day 28 ± 7

- Perform interim history and physical exam, emphasizing examination of any acute complaints.
- Record vital signs.
- Obtain blood for anti-AMA1 antibody ELISA.

6.1.11 Day 56 ± 3 (Day of Second Vaccination)

- Perform interim history and physical exam, emphasizing examination of any acute complaints.
- Record vital signs.
- Obtain blood for haematology, biochemistry, anti-AMA-1 antibody ELISA and exploratory immunology.
- Obtain a urine sample for to test for the presence of protein, glucose or blood.
- For females, perform a urinary β-hCG test. Ensure that test is negative before proceeding; a positive test will exclude the volunteer from receiving further vaccinations.
- Administer the vaccine.
- Observe for 30 minutes after vaccination to evaluate for immediate adverse reactions.
- Give subject oral thermometer, diary card and instructions for completion.

6.1.12 Day 59 ± 1 (3 days after Second Vaccination)

- Perform interim history and examine the injection site and any body systems felt to be necessary by the Investigator.
- Record vital signs.
- Review diary card.

6.1.13 Day 63 ± 2 (7 days after Second Vaccination)

- Perform interim history and examination of the injection site, and physical exam if indicated any acute complaints.
- Record vital signs.
- Obtain blood for haematology, biochemistry, anti-AMA-1 antibody ELISA
- Obtain a urine sample for to test for the presence of protein, glucose or blood.
- Collect Day 0-6 diary card.

6.1.14 Day 70 ± 3 (14 days after Second Vaccination and DAY OF CHALLENGE)

- Perform interim history and examine the injection site and any body systems felt to be necessary by the Investigator.
- Record vital signs.
- Insert cannula in forearm vein, obtain blood and flush cannula with normal saline.
- Obtain blood for haematology, biochemistry, dsDNA, anti-AMA-1 antibody ELISA, GIA, baseline PCR, and exploratory immunology.
- Obtain a urine sample for to test for the presence of protein, glucose or blood.
- For females, perform a urinary β-hCG test. Ensure that test is negative before proceeding; a positive test will exclude the volunteer from receiving challenge inoculum.
- For each volunteer insure that the inoculum is injected within 40 minutes of it being prepared. Flush cannula with normal saline again.
- Observe for 1 hour to evaluate for immediate adverse reactions, then remove cannula.

6.1.15 Day 71 (1 day after Challenge)

- Perform abbreviated history using malaria symptom questionnaire.
- Record vital signs.
- Obtain blood for blood smear and PCR.

6.1.16 Days 72 – Day 82 (2 - 12 days after Challenge)

Each day until a challenge endpoint is passed (i.e. malaria is diagnosed or day 16 post-challenge is reached), volunteers will be required to attend for two visits, approximately 12 hours apart. At each visit the following will be performed:

- Perform abbreviated history using malaria symptom questionnaire.
- Record vital signs.
- Obtain blood for blood smear and PCR.

6.1.17 Days 83 – Day 86 (13 – 16 days after Challenge)

Each day until a challenge endpoint is passed (i.e. malaria is diagnosed or day 16 post-challenge is reached), volunteers will be required to attend for one visit daily. At each visit the following will be performed:

- Perform abbreviated history using malaria symptom questionnaire.
- Record vital signs.
- Obtain blood for blood smear and PCR.

6.1.18 Day of Diagnosis – endpoint and anti-malarial therapy

At the point that a challenge endpoint has been reached, subjects will receive treatment with anti-malarial medication. Volunteers will be treated with oral Riamet®. This is a licensed drug in the UK for treatment of acute uncomplicated malaria caused by *Plasmodium falciparum* and has achieved a cure rate of 97.7% (Van Vugt et al 2000). It is a combination drug consisting of artemether (20 mg) and lumefantrine (120 mg) per tablet. A treatment course of Riamet® consists of 6 times 4 tablets. The first 4 tablets will be given when diagnosis is made, followed by additional doses after 8, 24, 36, 48 and 60 hours. Tablets should be taken

together with a meal. Treatment will be observed on three occasions at least and daily slide reading will be continued until two consecutive slides are negative for parasites. In the unlikely event of a treatment failure with Riamet, a second line of treatment (chloroquine) will be provided as follows: Volunteers will be treated with oral chloroquine, and will be treated as out-patients if they have mild or no symptoms. Chloroquine 150mg tablets will be administered as 600mg (4 tablets) orally at time 0, then 300mg (2 tablets) at 8 hours, 300mg (2 tablets) at 24 hours and 300mg (2 tablets) at 48 hours. The treatment administration will be directly observed by one of the investigators or the research nurse, for the doses at 0, 24 and 48 hours. (The infecting strain of malaria is known to be chloroquine sensitive, see Section 8.1.1.)

Volunteers who remain film negative at 16 days post-challenge will be considered protected from malaria but will still receive Riamet® treatment as a precaution. The course will be administered and observed as above.

The following will also be performed:

- For females, obtain a urine sample for β-hCG testing. If positive, consideration will be given to using an alternative treatment for malaria (chloroquine, as previously described) and advice will be sought from a local hospital infectious diseases consultant.

6.1.19 Day of Diagnosis +1, +2 – anti-malarial therapy

Volunteers will be required to attend once daily for at least two days following the day treatment has been commenced.

At each visit the following will be performed:

- Perform abbreviated history using malaria symptom questionnaire.
- Record vital signs.
- Obtain blood for blood smear and PCR.

After a positive blood film, blood samples will be collected daily until two consecutive negative thick smears are obtained.

6.1.20 Day 84 ± 3 (14 days after Challenge)

Volunteers will be required to attend on Day 84, whether or not they have already received anti-malarial treatment. At this visit the following will be performed:

- Perform abbreviated history using malaria symptom questionnaire.
- Record vital signs.
- Obtain blood for anti-AMA-1 antibody ELISA
- Obtain blood for blood smear and PCR (if scheduled as per Section 6.1.17 above)

6.1.21 Day 98 ± 7 Follow-up evaluation (28 days post-challenge)

- Perform interim history and examination of the injection site, and physical exam if indicated by acute complaints.
- Record vital signs.
- Obtain blood for haematology, biochemistry, anti-AMA-1 antibody ELISA, GIA and exploratory immunology.
- Obtain a urine sample for to test for the presence of protein, glucose or blood.

6.1.22 Day 140 ± 14 Follow-up evaluation (10 weeks post-challenge)

- Perform interim history and examination of the injection site, and physical exam if indicated by acute complaints.
- Record vital signs.
- Obtain blood for haematology, biochemistry

6.1.23 Day 238± 14 Final visit (24 weeks post-challenge)

- Perform interim history and physical examination.
- Record vital signs.
- Obtain blood for haematology, biochemistry, anti-dsDNA, HIV, HBV, HCV, anti-AMA-1 antibody ELISA,GIA and exploratory immunology.
- Obtain a urine sample for to test for the presence of protein, glucose or blood.

6.2 Minimisation of Bias

Laboratory staff analysing blood films for malaria and carrying out parasite PCR, ELISA, and GIA assays will be blinded to the group allocation of vaccinated volunteers and controls. Clinicians, volunteers and laboratory staff for other assays will not be blinded.

6.3 Study Vaccine

6.3.1 AMA1-C1/Alhydrogel®:

AMA1-C1/Alhydrogel® malaria vaccine is supplied as a slightly turbid suspension in single-dose vials for IM administration. Each 2.0 mL vial contains 1.0 mL. 0.5 mL of vaccine contains 800 µg Alhydrogel® (up to the equivalent of 400 µg of aluminium) onto which 80 µg of recombinant AMA1-C1 has been bound.

Both recombinant AMA1-FVO and AMA1-3D7 are highly purified 62 kilodalton (kDa) proteins that correspond to the ectodomain of the mature AMA1 protein from the FVO and 3D7 strains of *P. falciparum* respectively. In addition both have a 6-histidine C-terminal tag to allow purification of the protein. AMA1-FVO and AMA1-3D7 were purified from the fermentation supernatant using a combination of affinity, ionic, hydrophobic, and gel filtration chromatography. The purification process was designed to separate full-length product from degraded material as well as non-product-related impurities. Bulk Drug Substances were both manufactured at the WRAIR Bioproduction Facility (Silver Spring, Maryland) according to cGMP. The Drug Substances were prepared in unbuffered, isotonic saline without preservatives. The product conforms to the US Code of Federal Regulations for endotoxin, sterility, and general safety.

Aluminium hydroxide gel (Alhydrogel®, HCI Biosector, Denmark) has been extensively used as an adjuvant in many licensed human vaccines. Aluminium-containing adjuvants are in routine human use and contained in many licensed human vaccines.

6.3.2 CPG 7909

CPG 7909 is supplied in sterile single dose vials of 0.08 mL at 10 mg/mL in saline for IM administration. It is a short synthetic oligodeoxynucleotide of the following sequence: 5'-TCG TCG TTT TGT CGT TTT TTT CGA-3', with all nucleotides linked with phosphorothioate bonds. CPG 7909 is manufactured according to cGMP standards and conforms to the US Code of Federal Regulations for endotoxin, sterility, and general safety.

6.3.3 Vaccine Labelling

The two separate components of the vaccine will be separately labelled. Examples of the double-sided 'flag' label carrying required details for each component are below:

CLINICAL TRIAL: VAC035	FOR CLINICAL TRIAL USE ONLY
**AMA1-C1/Alhydrogel® VACCINE**	Sponsor: University of Oxford
1.0 mL (containing 160µg AMA1-C1)	CCVTM, Old Road, Oxford. OX3 7LJ
Vial No: ___ For Intramuscular Injection	Tel: 01865 857382
Batch no: BDP L0803002	Fax: 01865 857471
Volunteer no: ___ Store at +2 to 8°C	
Expiry Date: ____	

CLINICAL TRIAL: VAC035	FOR CLINICAL TRIAL USE ONLY
**CPG 7909**	Sponsor: University of Oxford
0.08 mL (containing 10mg/mL)	CCVTM, Old Road, Oxford. OX3 7LJ
Vial No: __ For Intramuscular Injection	Tel: 01865 857382
Batch no: BDP L08080002	Fax: 01865 857471
Volunteer no: ___ Store at +2 to 8°C	
Expiry Date: ____	

6.3.4 Preparation and Administration of AMA1-C1/Alhydrogel® + CPG 7909:

The trial site will prepare the point of injection formulation. Shortly before vaccination, 0.7 mL of AMA1-C1/Alhydrogel® is withdrawn and added to the single dose CPG 7909 vial. The vial, now containing 0.7 mL of AMA1-C1/Alhydrogel® and 0.08 mL of CPG 7909, is then mixed. The vaccine must be administered not more than 6 hours after mixing the AMA1-C1/Alhydrogel® with the CPG 7909 and should be kept at 2°C to 8°C if not immediately administered. When ready to administer AMA1-C1/Alhydrogel® + CPG 7909, 0.55 mL is withdrawn into a syringe and the vaccine is injected. A 0.55 mL dose of AMA1-C1/Alhydrogel® + CPG 7909 corresponds to 80 µg of AMA1-C1 and 564 µg of CPG 7909.

6.3.5 Vaccine Storage

AMA1-C1/Alhydrogel®, CPG 7909, and AMA1-C1/Alhydrogel®+CPG 7909 should be maintained at 2°C to 8°C until just prior to administration and should NOT be frozen at any time.

6.4 Duration of Study

Volunteers will attend for 2 vaccinations, administered two months apart, followed two weeks later by the malaria challenge. After the challenge there will be a period of intense follow up until the challenge endpoint is reached. After antimalarial treatment, volunteers will attend three further visits, one on day 98 (28 days following the challenge), one on day 140 (10 weeks following the challenge) and a final visit on day 238 (28 weeks following the challenge). Further follow-up of on-going adverse events or for any other reason, whether by visit or telephone, will be arranged at the last study visit if required.

6.5 Definition of End of Trial

The end of trial is the date of the last visit of the last patient to the study site.

6.6 Discontinuation Criteria

In accordance with the principles of the current revision of the Declaration of Helsinki (last amended November 2008) and any other applicable regulations, a volunteer has the right to withdraw from the study at any time and for any reason, without prejudice to his or her future medical care by the physician or at the institution, and is not obliged to give his or her reasons for doing so. If a subject withdraws after the challenge inoculum has been administered they must take a full course of anti-malarial medication and attend for two further safety follow up visits.

The Investigator may withdraw a volunteer at any time in the interests of their health and well-being. In addition a volunteer may withdraw/be withdrawn for any of the following reasons:

- Administrative decision by the Investigator
- Ineligibility (either arising during the study or retrospective having been overlooked at screening)
- Significant protocol deviation
- Patient/subject non-compliance with treatment regime or study requirements
- An adverse event which requires discontinuation of the study medication or results in inability to continue to comply with study procedures

The following adverse events associated with vaccine immunisation constitute absolute contraindications to further administration of vaccine. If any of these events occur during the study, the subject must be withdrawn and followed until resolution of the event, as with any adverse event.

- Anaphylactic reaction following administration of vaccine
- Pregnancy

The following adverse events constitute contraindications to administration of vaccine at that point in time; if any one of these adverse events occurs at the time scheduled for vaccination, the subject may be vaccinated at a later date, or withdrawn at the discretion of the Investigator. The subject must be followed until resolution of the event as with any adverse event.

- Acute disease at the time of vaccination

  Acute disease is defined as the presence of a moderate or severe illness with or without fever. All vaccines can be administered to persons with a minor illness such as diarrhoea or mild upper respiratory infection, with or without low-grade febrile illness, *i.e.*, temperature of <37.5°C/99.5°F.

- Temperature of ≥37.5°C (99.5°F) at the time of vaccination

6.7 Accountability of the Study Treatment

6.7.1 Vaccine Supply

The AMA1-C1/Alhydrogel® and CPG 7909 for this protocol will be supplied to the study site by the Biopharmaceutical Development Program (BPD), Science Applications International Corporation (SAIC), Frederick, MD where the materials were formulated and vialed. Both will be supplied in single-use vials labelled for investigational use only.

The AMA1-C1/Alhydrogel® vaccine, as well as CPG 7909, will be transported to the UK under temperature controlled conditions where temperature recording devices will accompany the vaccines at all times to ensure storage temperature limits have not been violated. Vaccine will be stored at the CCVTM, Churchill Hospital, Oxford, UK in a refrigerator at 2°C to 8°C; refrigerator temperature will be monitored continuously.

6.7.2 Disposition of Used/Unused Supplies

After administration of a vaccine dose, the used vials will be disposed of according to the standard operating procedure (SOP 07). At the conclusion of the study, all unused AMA1-C1/Alhydrogel® and CPG 7909 vaccine supplies will be returned to Malaria Vaccine Development Branch (MVDB). Unused vaccine supplies will be transported under temperature controlled conditions with temperature recording devices.

The Investigator is responsible for maintaining an accurate inventory and accountability record of vaccine supplies for this study. Partially used vials of AMA1-C1/Alhydrogel® or CPG 7909 may not be administered to other volunteers.

6.8 Randomisation

Subjects will not be randomised to groups. Neither subjects nor clinicians will be blinded. However, laboratory staff analysing blood films for malaria and carrying out parasite PCR assays, ELISA, and GIA will be blinded to the study treatment group. Laboratory staff performing other immunologic analyses may not be blinded.

6.9 Source Data

Source documents are original documents, data, and records from which the subject's CRF data are obtained. For this study, these will include, but are not limited to, volunteer consent form, blood results, GP response letters, laboratory and drug accountability records, diaries, and correspondence.

In the majority of cases, CRF entries will be considered source data as the CRF is the site of the original recording (i.e., there is no other written or electronic record of data). In this study this will include, but is not limited to vital signs, physical examination records and measurements, urine pregnancy assessments, adverse event data and details of vaccinations and malaria challenge.

All source data and subject CRFs will be stored securely (see section 14.5).

7. TREATMENT OF TRIAL SUBJECTS

7.1 Name and description of investigational products

See section 6.3.

7.2 Known and potential risks and benefits

The risks associated with malaria infection are described in Section 8.2. Risks to the volunteers from vaccination are those associated with venepuncture and with immunisation. These risks are outlined below.

Female participants will be cautioned of the unknown risk of the AMA1-C1/Alhydrogel® + CPG 7909 vaccine to the fetus and will be advised to use adequate birth control methods for at least two weeks before immunisation and for the duration of the study.

7.2.1 Venepuncture

Risks occasionally associated with venepuncture include pain and bruising at the site of venepuncture, light-headedness, and syncope (rarely).

7.2.2 Immunisation with AMA1-C1/Alhydrogel®

Possible local vaccine reactions include pain, swelling, erythema, induration, limitation of limb movement for several days, lymphadenopathy, or pruritis at the injection site. Local subcutaneous nodules, believed to be granulomatous reactions to aluminium hydroxide, have been observed with use of aluminium hydroxide-based adjuvants. Thus, most aluminium hydroxide-adsorbed vaccines are injected intramuscularly rather than subcutaneously. Systemic reactions such as fever, chills, headache, fatigue, malaise, myalgia, joint pain, may also possibly occur. Immediate hypersensitivity reactions including urticaria, anaphylaxis, or other IgE-mediated responses are possible as with any vaccine. As with any investigational vaccine, there is a theoretical possibility of risks about which we have no present knowledge. Volunteers will be informed of any such risks should further data become available.

7.2.3 Immunisation with CPG 7909

CPG 7909 is a potent immune activator, and has the theoretical potential to overcome the normal tolerance of the immune system for self antigens and induce autoimmune disease. In a number of murine models, administration of CPG 7909 has been associated with development of certain autoimmune phenomena. However, the relevance of this finding to humans is unclear because while TLR-9 receptors are widely distributed among immune cells in mice, they appear to be restricted to B cells and plasmacytoid dendritic cells in humans. [29*Mali] While subjects in several studies using CPG 7909 have developed detectable antibody to dsDNA, no subject in any study to date has developed signs or symptoms of a lupus-like autoimmune disease. Anti-dsDNA antibodies have generally occurred after multiple vaccinations, but declined to baseline after termination of vaccination. The clinical significance of the dsDNA antibody elevations is unknown. One patient in a study investigating immune therapy for treatment of melanoma exhibited elevated antimicrosomal antibodies while receiving CPG 7909 along with peptides and Montanide ISA 51. The patient, who had a known history of sub-clinical enlargement of the thyroid gland with borderline insufficiency of thyroid function associated with elevated TSH and no clinical symptoms, had a long-term exposure to anticancer immune therapy prior to CPG 7909 treatment. Pre-CPG 7909 treatment blood levels of antimicrosomal antibodies were not assayed. No other patients who have received CPG 7909 are known to have developed autoimmune disease. Subjects in this study will be closely monitored for laboratory evidence of autoimmunity by following urine for protein and blood (as a marker of autoimmune

nephritis). Serum will be collected for anti-dsDNA testing. Clinical signs or symptoms of autoimmunity will also be followed closely.

Phase 1 studies of CPG 7909 have shown fluctuations in hematologic indices (an initial rise, then decrease in total leukocytes, neutrophils, and lymphocytes, followed by a return to baseline between 7 to 15 days) at all dose levels. Decreases in WBC counts at Day 3, followed by a return to baseline by Day 7, were seen in Phase 1 studies with CPG 7909 as a vaccine adjuvant (Section 3.2). No clinical events have been associated with these changes. A Phase 3 clinical study of CPG 7909 in patients with advanced nonsquamous cell lung cancer showed a possible increase in sepsis and thrombocytopenia with bleeding events in volunteers receiving CPG 7909. Volunteers with acute febrile illness will be excluded from vaccinations and hematologic indices will be followed after each vaccination.

Other clinical studies of CPG 7909 used as an adjuvant have shown a moderately increased incidence of mild to moderate local and systemic AEs after vaccination (injection site pain, myalgia, fatigue). See Section 3.2.3**.** These AEs will be monitored and recorded as per standard practice.

Maternal and fetal toxicity were noted in pregnant rabbits and rats that were administered CPG 7909 during the period of organogenesis. External, visceral, and skeletal malformations of the fetus were noted as well as reduction in maternal weight, body weight gains, and food consumption. Female participants will be cautioned of the unknown risk of the AMA1-C1/Alhydrogel®+CPG 7909 vaccine to the fetus, and will be advised to use adequate birth control methods for at least 2 weeks before immunization and for the duration of the trial. Any female participant interested in contraceptive methods will be referred to the local health center family planning services for evaluation and institution of an appropriate contraceptive method.

7.2.4 Benefits

Study participants may not receive any direct benefit from participation in this study. It is hoped that information gained in this study will contribute to the development of a safe and effective malaria vaccine.

All subjects will be entitled to full UK National Health Service (NHS) care, free of charge, for any medical complication arising during the course of the trial. Investigators are able to make a referral to the appropriate specialty or to a volunteer's GP with their consent.

7.3 Precautions to minimise risk

7.3.1 Immunisation

As outlined above, the participants will be monitored closely during their participation in this study. The study vaccines have been produced according to current Good Manufacturing Procedures (GMP). The vaccines will be administered by experienced Investigators with drugs and equipment available for the treatment of anaphylaxis and other potential adverse reactions. All vaccine doses will be given by IM injection to minimize injection site reactions such as pain.

7.3.2 Protection of Study Staff

All study personnel have been trained to follow Universal Precautions. Clinical procedures will be carried out in accordance with CCVTM SOPs including SOP-MC006 (Vaccination) and SOP-MC007 (Venepuncture).

7.4 Dosage and route of administration

All volunteers in group 1 will receive two injections of AMA1-C1/Alhydrogel® in CPG 7909, separated by two months. All doses will be given by IM injection in the deltoid muscle in alternating arms. The doses to be used in this study will be: AMA1-C1: 80 µg, Alhydrogel®

800 µg (aluminium: 400 µg), CPG 7909 ODN: 564 µg. This dose has been used in clinical trials in the US and no significant safety issues have been identified.

Group 2 will consist of unvaccinated control volunteers to be included in the challenge phase of the study.

7.5 Concomitant Medication

Subjects should avoid taking any drugs or medications (prescription and/or over-the-counter) where possible. Any such medicine will be recorded in the CRF and the Investigator will review on-going eligibility of the subject. The Investigators may prescribe any concomitant medications or treatments deemed necessary to provide adequate supportive care, these include analgesics and anti-emetic medication for use during malaria infection.
Any medication, other than the investigational vaccines and antimalarial medication, that is taken during the study will be recorded in the CRF.

7.6 Compliance with Dosing Regime

All doses of investigational vaccine in this two dose study will be administered by the Investigator and recorded in the CRF. The study vaccine will never be in the possession of the subject and compliance will not therefore be at issue. Subjects will be provided with anti-malarial medication at the point of diagnosis. Where Riamet is used, at least 3 of the 6 doses will be directly observed by the Investigator to ensure compliance.

8. MALARIA CHALLENGE

8.1 Background

The samples we propose to use as infectious inocula were produced by Drs Gregor Lawrence, Allan Saul and colleagues at the Queensland Institute of Medical Research (QIMR) in Brisbane, Australia in 1994. The protocol for the study was reviewed and approved by the QIMR Ethics Committee and the Healthy Volunteer Studies Research Ethics Subcommittee, Lothian Health Board (Edinburgh). Procedures were designed to minimise the risk of other infectious agents in the cryopreserved samples. The cryopreserved *P. falciparum* has been made available to CCVTM under a collaborative research agreement.

8.1.1 Collection and cryopreservation of inoculum

Laboratory-reared *Anopheles stephensi* mosquitoes were infected with the *P. falciparum* clone 3D7 (a chloroquine-sensitive strain) by membrane feeding on a blood meal containing gametocytes. Ten and fourteen days later, the mosquitoes were fed on two volunteers. Parasitaemia in the volunteers was followed by daily microscopy from day 4 after infection. Blood was taken from the volunteers 6 hours after they developed fever, when both were microscopically parasite positive. The volunteers were treated with chloroquine soon after blood was drawn with complete recovery.

The inoculum was prepared as detailed by Cheng et al 1997. Blood was collected at the Australian Red Cross Blood Bank in an aseptic manner using standard blood bank equipment. The leukocytes were removed with a leukocytic filter. The thawing and washing of the cells reduced the amount of serum transferred with the red cells by a factor of 1000, compared to injecting the same volume of blood. The volume of inoculum to be given to each volunteer contains a very small volume of red blood cells, equivalent to only 1.5 to 4 micro litres of blood.

The red cells were cryopreserved using a protocol from the American Association of Blood Banks Technical Manual that is normally employed for freezing blood from patients and donors with rare blood groups. Blood from both volunteers was group O and Rhesus negative.

Viability of the inoculum was confirmed by culture in December, 2006, and will be reconfirmed prior to initiation of the study. Viability up to the time expected to be required for inoculations (40 minutes) will also be confirmed at that time. For each vial of inoculum used a small amount will be cultured after inoculations are complete to confirm viability and the estimated number of parasitized red blood cells inoculated.

8.2 Known and potential risks

8.2.1 Malaria

The risks of participation in this study are very low providing that all subjects return for follow up as outlined. If untreated, the malaria infection that we propose to give could result in death. Worldwide over 1000 people have been experimentally infected with malaria and all have made a complete recovery. In Oxford 240 people have taken part in malaria challenge studies. Most, but not all, of the people who have participated in these studies have experienced some of the symptoms of malaria such as fevers, chills, muscle pains, headaches or fatigue, generally lasting 1 to 3 days. Some volunteers may feel tired and lethargic after malaria infection. Less likely is dizziness, fainting or vomiting. Severe problems are unlikely. Two volunteers in Oxford have required hospital admission in the past for observation. No persistent problems have been noted. Painkillers such as paracetamol and anti-emetics will be prescribed if required.

There has been one serious adverse event previously in a person infected in a malaria sporozoite, rather than blood stage, challenge study in continental Europe, in which a volunteer developed chest pain in association with raised serum cardiac troponin two days

after completion of malaria treatment, (lumenfantrine / artemether - Riamet®). It is uncertain whether this was due to coronary artery spasm or myocardial inflammation. It is also unclear whether it related to infection with malaria, malaria treatment or an alternative cause. In response to this event we will therefore exclude from the study volunteers with: -

- An estimated, ten year risk of fatal cardiovascular disease of ≥5%, as estimated by the Systematic Coronary Risk Evaluation (SCORE) system (Conroy et al 2003)
- A previous medical history of cardiac arrhythmia or prolonged QT interval;
- A family history of sudden cardiac death

The less commonly used NF54 strain of *Plasmodium falciparum* was used for sporozoite challenge in this trial as opposed to the 3D7 strain used safely in over 1000 volunteers worldwide including 240 volunteers in Oxford.

8.2.2 Venepuncture

Risks occasionally associated with venepuncture include pain and bruising at the site of venepuncture, light-headedness, and syncope (rarely).

8.2.3 Blood-borne infection from inoculum

The major issue to be addressed with a blood stage challenge system is the risk of other infectious agents in the malaria-infected blood. This question was addressed by testing the donors extensively for possible infections before they were infected with malaria and for 30 months after donation.

The volunteers were healthy at the time of malaria infection and had normal full blood counts and liver function tests. In the three months following malaria infection, both volunteers recorded their temperatures daily and remained afebrile. Infections tested for included syphilis, hepatitis A, B and C, human T lymphotropic virus type 1, human immunodeficiency virus and Ross River virus. As both volunteers were Epstein Barr virus (EBV) and cytomegalovirus (CMV) seropositive, only volunteers seropositive for these two common pathogens will be considered for this study.

The risk of transmission of variant Creutzfeldt Jacob Disease (vCJD) from the inoculum appears remote. The two donors are Australian residents, where no cases of either Bovine Spongiform Encephalopathy (BSE) in cattle or vCJD in humans have been reported to date.

8.3 Precautions to minimise risk from malaria

The risk posed by malaria infection will be minimised by close follow up at the Churchill Hospital in Oxford followed by prompt treatment with a full course of antimalarial medication as soon as a single parasite is detected on microscopy of a subject's thick film. Subjects will attend once or twice daily after the challenge day for medical assessment and two blood tests for malaria (thick film analysis and PCR). If the Investigator is concerned about a subject but the blood film is negative, the subject may be commenced on antimalarial medication based on a positive PCR result and typical symptomatology.

If needed, a trial physician can be contacted directly by volunteers 24 hours a day throughout the trial. Although hospital admission is not planned or expected, if clinical concerns arise, the Investigator may arrange admission to the on-site NHS infectious diseases unit for independent assessment and management under a specialist Consultant physician.

All volunteers will be counselled as to the risks of malaria and will be required to be resident near the trial centre for the duration of the challenge phase. The current WHO-recommended antimalarial medication, Riamet®, will be used to eradicate parasites after diagnosis, unless

© The University of Oxford 2010

contraindicated. The subjects will continue to have daily blood films until two consecutive thick blood films are negative.

8.4 Previous experimental use of malaria-infected red blood cells

Previous experience with the inoculum to be used in this study is summarized in Section 3.2.6; no additional risk associated with the inoculum has been identified to date.

8.5 Storage conditions

Between 1994 and 2003 the cryopreserved samples to be used in this trial were stored in dedicated liquid nitrogen cylinders in a secure facility at QIMR. The liquid nitrogen containers were kept locked and accessible only to approved staff. In 2003 the samples were transferred to Biotec Distribution Ltd., Bridgend, UK and then to Thermo Fisher Bishop's Stortford, Hertfordshire, UK., in 2007 where they have been stored on behalf of Oxford University in temperature-monitored liquid nitrogen since.

8.6 Administration of the Inoculum

8.6.1 Preparation

Thawing and washing of the inoculum will be done with commercial solutions for human use and with disposable syringes and needles according to standard operating procedures used in previous studies at QIMR and Oxford. Work will be carried out in the bio safety category III laboratory at the Jenner Institute, ORCRB Sample manipulations will be performed within a safety cabinet that has been especially cleaned and set aside for this purpose.

After thawing of the blood inoculum, the cold chain (2-8 degrees) must be maintained at all times until the inoculum has been administered to the volunteer.

8.6.2 Administration

The inoculation will take place in Oxford at the CCVTM. The Inoculum will be administered by intravenous injection into an indwelling intravenous cannula. Approximately 1,000 infected red blood cells will be injected in a total volume of 5 mL of normal saline followed by a saline flush. Subjects will be observed for one hour before discharge. The order in which vaccinated and unvaccinated volunteers receive the inoculum will be interspersed in case of time effects on viability of the parasites. If the inoculum is given on more than one day an equal number of control volunteers will receive the inoculum on each day. All volunteers will receive inoculum within 40 minutes of preparation.

9. OUTCOME MEASURES

Outcome measures for this study will include the following:

- Levels of parasite growth inhibitory activity measured in laboratory assays
- Rate of parasite multiplication (measured by sequential PCR assays for malaria)
- Safety data accrued during the study
- Immunological assays of response to vaccination and malaria challenge (anti-AMA1 antibody responses)
- Time to detection of a malaria parasite on the thick blood film

9.1 Levels of Parasite Growth Inhibitory Assay (GIA)

Serum samples will be collected from volunteers at several points during the study (see flowchart, Appendix A). At least 7 mL of serum from each volunteer at each time point will be shipped to MVDB, USA for GIA.

The GIA is designed to determine whether antibodies obtained from an immunized person can inhibit the process of merozoite invasion into red cells. In this assay, synchronized blood-stage parasites from the FVO and 3D7 lines are incubated with purified IgG from volunteer sera for a period of 40 hours *in vitro*. During this period, merozoites emerge from the infected red cells, invade normal red cells, and initiate a new growth cycle. The percentage reduction in the number of newly invaded red cells in the presence of the post vaccination IgG compared to the number of newly invaded red cells in the presence of the day zero purified IgG is calculated as the growth inhibitory activity.

9.2 Parasite Multiplication Rates

Blood samples will be collected once or twice daily after the challenge for Quantitative PCR measurement of parasite numbers according to CCVTM SOP-ML008. Using this information, an estimate of parasite growth rate will be obtained as described by Bejon (Bejon et al., 2005)

9.3 Safety data

Safety data will be collected at each study visit, as described in Section 10.

9.4 Immunological Assays

The following parameters will be considered evidence of the impact of vaccination in inducing malaria-specific immune responses.

- **Antibody response.** Serum collected at designated visits (see flowchart, Appendix A) will also be used for antibody measurement. ELISA will be used to assess the levels of anti-AMA1 antibodies induced by vaccination. ELISA studies will be done at MVDB, USA.
- **Other immunologic responses.** Exploratory immunology will be performed at the University of Oxford on one or more of the following assays:
  1) Interferon gamma AMA-1 peptide ELISPOT.
  2) Flow cytometry to measure T cell responses to AMA-1
  3) B cell ELISPOT on plasma and B cells
  4) Cultured interferon gamma AMA-1 peptide ELISPOT

9.5 Parasite Identification by Thick Film Analysis

Thick films will be prepared according to the CCVTM SOP-ML009 and analysed by an experienced slide reader. The presence of a single *Plasmodium falciparum* parasite on thick film microscopy will be the point of diagnosis of malaria parasitaemia. The time in hours between malaria challenge and a parasite positive thick film will be calculated for each individual.

10. ASSESSMENT OF SAFETY

Safety will be assessed by the frequency, incidence and nature of adverse events and serious adverse events arising during the study.

10.1 Safety Parameters

Safety parameters will include diary cards which record symptoms from days 0-6 following each vaccine; vital signs and any physical examination performed at study visits; recording of daily oral temperature for 6 days following vaccination; laboratory studies and unsolicited adverse events.

10.2 Methods and Timing of Safety Assessments

Subjects will be asked to keep daily symptom diaries recording oral temperature once daily, as well as pain/tenderness, redness, induration, warmth and itch at the injection site for six days following each immunisation. The size of any injection site reaction will be measured utilizing a standardized measurement device and recorded in the volunteer symptom diary. All volunteers must wait at the trial centre for a further assessment 30 minutes after vaccination in case of severe allergic reaction to the vaccine.

Using standard techniques, the clinical laboratory, will perform the following tests at designated visits (see flowchart, Appendix A):

1. Full blood count (parameters to be assessed for safety: WBC, platelet count and haemoglobin)
2. Serum sodium, potassium, urea, and creatinine
3. Bilirubin, albumin, total protein, alanine aminotransferase (ALT), and alkaline phosphatase (ALP)
4. anti-dsDNA

Urine ß-hCG testing and analysis for protein, blood and glucose will be performed at the clinical trial site, using appropriate licensed test kits.

The grading scales for clinical reactions are given in Appendix C, D, and E.

10.3 Definitions

10.3.1 Adverse Event (AE)

An AE or adverse experience is:

'Any untoward medical occurrence in a patient or clinical investigation subject administered a medicinal product, which does not necessarily have to have a causal relationship with this treatment (the study medication)'.

An AE can therefore be any unfavourable and unintended sign (including an abnormal laboratory finding), symptom or disease temporally associated with the use of the study medication, whether or not considered related to the study medication.

10.3.2 Adverse Drug Reaction (ADR)

All untoward and unintended responses to a medicinal product related to any dose.

The phrase "responses to a medicinal product" means that a causal relationship between a study medication and an AE is at least a reasonable possibility, i.e., the relationship cannot be ruled out.

10.3.3 Unexpected Adverse Reaction

An adverse reaction, the nature or severity of which is not consistent with the applicable product information (e.g. Investigator's Brochure or summary of product characteristics).

10.3.4 Expected Adverse Drug Reactions

An adverse reaction, the nature or severity of which is consistent with the applicable product information (e.g. Investigator's brochure or summary of product characteristics).

No significant safety concerns have been identified in the initial trials of AMA1-C1/Alhydrogel® or AMA1-C1/Alhydrogel® + CPG 7909 (see also section 3.2.1). Preclinical testing has also shown no safety issues, as detailed in the Investigator's Brochure.

Expected AEs for AMA1-C1/Alhydrogel® include pain, swelling, erythema, induration, limitation of limb movement for several days, lymphadenopathy and pruritis at the injection site. Systemic reactions such as fever, chills, headache, fatigue, malaise, myalgia and arthralgia may also possibly occur. Immediate hypersensitivity reactions including urticaria, anaphylaxis, or other IgE-mediated responses are possible as with any vaccine.

Use of CPG 7909 as an adjuvant may increase local reactogenicity and systemic symptoms such as fatigue or myalgia. Transient fluctuations in neutrophils and total WBC counts may also occur. The risk of autoimmune disease is theoretical at this point, but clinical and laboratory markers for autoimmune disease will be followed, and persons with pre-existing autoimmune disease should be excluded from clinical trials until more data are available (see section 7.2.3). As with any vaccine, immediate-type hypersensitivity reactions may occur, and volunteers should be monitored for 30 minutes following each immunisation to observe for anaphylactic responses.

Female participants will be cautioned of the unknown risk of study vaccines to the fetus and of animal fetal toxicity studies using high doses of CPG 7909 (Section 7.2.3), and will be advised to use adequate birth control methods for at least two weeks prior to enrolment and for the duration of the study.

10.3.5 Expected Symptoms of Malaria

The following symptoms of malaria are expected to occur in some or all of the volunteers in this study and will be recorded on the CRF but not routinely reported as AEs:

- Fever, feverishness, chills, rigors, sweats
- Headache, anorexia, nausea, vomiting, diarrhoea
- Arthralgia, myalgia, back pain

10.3.6 Serious or Severe Adverse Events

To ensure no confusion or misunderstanding of the difference between the terms "serious" and "severe", which are not synonymous, the following note of clarification is provided:

The term "severe" is often used to describe the intensity (severity) of a specific event (as in mild, moderate, or severe myocardial infarction); the event itself, however, may be of relatively minor medical significance (such as severe headache). This is not the same as "serious," which is based on patient/event outcome or action criteria usually associated with events that pose a threat to a patient's life or functioning. Seriousness (not severity) serves as a guide for defining regulatory reporting obligations.

10.3.7 Serious Adverse Event or Adverse Drug Reaction

A serious adverse event (experience) or reaction is any untoward medical occurrence that at any dose:

- Results in death,
- Is life-threatening,

NOTE: The term "life-threatening" in the definition of "serious" refers to an event in which the patient was at risk of death at the time of the event; it does not refer to an event which hypothetically might have caused death if it were more severe.

- Requires inpatient hospitalisation or prolongation of existing hospitalisation,

- Results in persistent or significant disability/incapacity, or
- Is a congenital anomaly/birth defect.

Medical and scientific judgment should be exercised in deciding whether an adverse event is serious in other situations.

10.3.8 Causality Assessment

All AEs will have their possible relationship to study vaccine assessed by an experienced Investigator using the following terms:

- **Definite**: Clear-cut temporal association, and no other possible cause.
- **Probable**: Clear-cut temporal association and a potential alternative aetiology is not apparent.
- **Possible**: Less clear temporal association; other aetiologies also possible.
- **Remote**: Temporal association between the AE and the vaccine or the nature of the event is such that the vaccine is not likely to have had any reasonable association with the observed illness/event (cause and effect relationship improbable but not impossible).
- **Not Related**: The AE is completely independent of vaccine administration; and/or evidence exists that the event is definitely related to another aetiology.

The degree of certainty with which an AE can be attributed to administration of the study vaccine will be determined by how well the event can be understood in terms of one or more of the following:

- The event being temporally related with vaccination or reproduced on re-vaccination.
- A reaction of similar nature having previously been observed with this type of vaccine and/or formulation.
- The event having often been reported in the literature for similar types of vaccines.

All local (injection-site) reactions will be considered causally related to vaccination.

10.4 Serious Adverse Event Reporting Procedures

All SAEs must be reported to the sponsor/Chief Investigator (CI) and to MVDB within one working day of discovery or notification of the event. All SAE information must be recorded on an SAE form and faxed or emailed to the sponsor/CI and MVDB. Additional information received for a case (follow-up or corrections to the original case) need to be detailed on a new SAE form and faxed or emailed to the sponsor and MVDB.

The sponsor/CI will report all suspected adverse reactions which are both serious and unexpected (SUSARs) to the Competent Authority (MHRA) and the Ethics Committee concerned. Fatal or life-threatening SUSARs must be reported within 7 days and all other SUSARs within 15 days. The sponsor/CI will also inform all Investigators concerned of relevant information about SUSARs that could adversely affect the safety of subjects.

In addition to the expedited reporting above, the sponsor/CI shall submit once a year throughout the clinical trial or on request a safety report to the Competent Authority and Ethics Committee.

SAEs will be reported to the NIAID Institutional Review Board (IRB) by the MVDB investigator as per NIAID IRB requirements. MVDB will also report adverse events to Coley Pharmaceutical Group as per the Pharmacovigilance Agreement between MVDB and Coley. Adverse events will be summarized at least annually and reported to the Regulatory Compliance and Human Subjects Protection Branch (RCHSPB)/NIAID and will be compiled in an annual report to the FDA and to the MHRA.

© The University of Oxford 2010

10.5 Reporting Procedures for All Adverse Events

All AEs occurring during the study observed by the Investigator or reported by the patient, whether or not attributed to study medication, will be reported on the CRF. AEs considered related to the study medication by the medical advisor or the sponsor will be followed where possible until resolution or the event is considered stable. The following attributes must be assigned by the Investigator: description, date of onset and resolution date, severity, assessment of relatedness to study medication, other suspect drug or device and action taken. The Investigator may be asked to provide follow-up information.

All related AEs that result in a patient's withdrawal from the study or are present at the end of the study, should be followed up where possible until a satisfactory resolution occurs.

All deaths occurring during the study must be reported to the Sponsor/CI and MVDB. These include deaths within 30 days of the final dose of study medication and deaths up to the last formal follow-up observational period, whichever is longer. For all deaths, available autopsy reports and relevant medical reports will be made available for reporting to the relevant authorities.

It will be left to the Investigator's clinical judgment whether or not an AE is of sufficient severity to require the patient's removal from treatment. A patient may also voluntarily withdraw from treatment due to what he or she perceives as an intolerable AE. If either of these occurs, the patient must undergo an end of study assessment and any appropriate medical care should be arranged.

The severity of events will be assessed according to the scales in Appendix C, D, and E. The relationship of AEs to the study medication will be assessed as detailed in section 10.3.8.

Any pregnancy occurring during the clinical study and the outcome of the pregnancy, should be reported to the sponsor and MVDB.

10.6 Local Safety Committee (LSC)

The study will have an appointed Local Safety Committee (LSC), which is composed of on-site Consultants. The Consultants will have experience in infectious diseases at the Oxford Radcliffe Hospitals NHS Trust, but are independent from the study and can review events in 'real time'. All severe (grade 3) adverse events (AE) not considered to be related to the challenge and any serious adverse events (SAE: see section 10.3.6 for definition) will be reported to one of the Committee members within 24 hours of the Investigators' knowledge of the event. The LSC members will be empowered to convene an Independent Advisory Committee (IAC) if deemed necessary. The LSC role will include:

- Provision of advice to the Investigators and the sponsor on whether a set of clinical circumstances in a study warrants formal notification of the IAC.
- Provision of clinical advice on any illness in study subjects, especially in circumstances in which treatment might influence the course of the study
- Withdrawal of individual subjects from the study if deemed to be necessary because of any severe (grade 3) or serious adverse event (SAE).
- Review and evaluate the safety data as needed and make recommendations to the Investigators concerning the continuation, modification or termination of the study.

10.7 Stopping Criteria

If a dose of vaccine is considered unacceptably reactogenic, additional vaccinations will be suspended until reviewed with the LSC, CI/study sponsor, and MVDB. The communications from the LSC will subsequently be forwarded by the investigators to the IEC and to the NIAID IRB. The following criteria will be used to define unacceptable reactogenicity of the AMA1-C1 malaria vaccines:

1. One or more volunteers experience an SAE (as defined in Section 10.3.7 in this protocol) that is determined to be possibly, probably, or definitely related to the vaccine (as defined in Section 10.3.9 in this protocol), **OR**

2. One or more volunteers experience a hypersensitivity reaction that is probably or definitely related to the vaccine, excluding a Grade 1 or 2 rash, **OR**
3. Any severe clinical illness occurs that is not explained by a diagnosis that is unrelated to vaccination, **OR**
4. Two or more volunteers experience a Grade 2 or higher laboratory abnormality, or Grade 3 systemic AE that is determined to be possibly, probably, or definitely related to the vaccine, as defined in Section 10.3.9 in this protocol. This does not include Grade 2 neutropenia resolving within 7 days of vaccination, which is an expected laboratory abnormality with CPG 7909 and which has not been associated with clinically significant events.

11. STATISTICS

11.1 Description of Statistical Methods

The primary purpose of the study is to measure the correlation between *in vitro* growth inhibition assay (GIA) measured at Day 70 (day of challenge) and multiplication rate *in vivo* using the method described in Bejon et al (2005). This calculation will be performed in SPSS software, by calculating Spearman's correlation coefficient and associated confidence intervals and/or significance tests. The secondary objective is to detect a difference in parasite growth rates between vaccinated volunteers and controls. The Wilcoxon-Mann-Whitney test will be used to determine statistical significance of any difference detected. Tertiary objectives are to assess the safety and reactogenicity of the study vaccine, and to assess immunogenicity in response to vaccination. The frequency of systemic and local adverse events will be summarized by severity and relationship to vaccine. Antibody titers determined by ELISA will also be summarized. Analysis of adverse events and immunogenicity will be descriptive.

11.2 The Number of Subjects

The magnitude of the effect on the vaccine on *in vivo* parasite multiplication rates, if any, is unknown. For the purposes of power calculations, we assume that the vaccinated subjects have GIA responses uniformly distributed from 0 to 100% and that the distribution of GIA for the control subjects has fewer than 5% of GIA responses greater than 10%. A table showing the power (rounded to the nearest percent) of the study to show that the rank correlation is larger than some value (denoted by different columns) given the true rank correlation is some other value (denoted by different rows). For example, the table shows that with a true rank correlation of .7 we have a power of about 84 percent to show that the rank correlation is greater than .5. This power is for a total sample size of 17 (5 control subjects and 12 vaccinated subjects). We have included 15 vaccinated subjects in case of losses to follow up prior to the malaria challenge, and have added one control subject (to a total of 6) so that if volunteers are inoculated over two days there will be an equal number of control subjects receiving inoculum on each day.

True Rank Correlation	Power>0	Power>.2	Power>.5	Power>.7
0.5	100	94	49	11
0.7	100	99	84	40
0.9	100	100	100	93
0.95	100	100	100	99

11.3 The Level of Statistical Significance

The level of significance used is >0.05.

© The University of Oxford 2010 CONFIDENTIAL

12. DIRECT ACCESS TO SOURCE DATA/DOCUMENTS

The Investigator(s)/institution(s) will permit trial-related monitoring, audits, IRB/IEC review, and regulatory inspection(s), providing direct access to source data/documents to authorised auditors and monitors.

© The University of Oxford 2010

13. QUALITY CONTROL AND QUALITY ASSURANCE PROCEDURES

Data will be evaluated for compliance with the protocol and accuracy in relation to source documents. The study will be conducted in accordance with procedures identified in the protocol. SOPs will be used at all clinical and laboratory sites. Regular monitoring will be performed according to the requirements of the Medicines for Human Use (Clinical Trial) Regulations 2004 and ICH Good Clinical Practice (GCP). Following written standard operating procedures, the monitors will verify that the clinical trial is conducted and data are generated, documented and reported in compliance with the protocol, GCP and the applicable regulatory requirements. The investigator site will provide direct access to all trial related source data/documents and reports for the purpose of monitoring and auditing by the sponsor, and inspection by local and regulatory authorities.

© The University of Oxford 2010 **CONFIDENTIAL**

14. ETHICS

14.1 Declaration of Helsinki

The Investigator will ensure that this study is conducted according to the principles of the current revision of the Declaration of Helsinki (last amended November 2008,).

14.2 ICH Guidelines for Good Clinical Practice

The Investigator will ensure that this study is conducted in full conformity with relevant regulations and with the ICH Guidelines for Good Clinical Practice (CPMP/ICH/135/95) July 1996.

14.3 Informed Consent

Written and verbal versions of Informed consent will be presented to the subject detailing no less than: the exact nature of the study; the implications and constraints of the protocol; the known side effects and any risks involved in taking part. It will be clearly stated that the patient/subject is free to withdraw from the study at any time for any reason without prejudice to future care, and with no obligation to give the reason for withdrawal.
The patient/subject will be allowed as much time as wished to consider the information, and the opportunity to question the Principal Investigator, their GP or other independent parties to decide whether they will participate in the study. Written Informed Consent will then be obtained by means of subject dated signature and signature of the person who presented informed consent. The original signed form will be retained at the study site. A copy or second signed original of the signed Informed Consent will be given to the subject.

14.4 Independent Ethics Committees

A copy of the protocol, proposed informed consent form, other written patient/subject information and any proposed advertising material will be submitted to an Independent Ethics Committee (IEC), Oxrec A (nres) for written approval.

The Investigator will submit and, where necessary, obtain approval from the IEC for all subsequent protocol amendments and changes to the informed consent document.
The Investigator will notify deviations from the protocol or SAEs occurring at the site to the sponsor and will notify the IEC of these where necessary and in accordance with local procedures.

The NIAID IRB will also review the protocol, informed consent, and written patient/subject information, and prior approval will be obtained from the NIAID IRB for all proposed amendments and changes to the informed consent. The MVDB investigator will notify the NIAID IRB of all protocol violations and SAEs in accordance with IRB guidelines.

14.5 Patient/subject Confidentiality

The Investigator will ensure that the subject’s anonymity is maintained. All documents will be stored securely, in confidential conditions and kept in compliance with the Data Protection Act. A unique study number will be the only volunteer identifier used on all trial records except where identifiable information is absolutely necessary (e.g. correspondence with GP or other third party with the volunteer’s consent, consent form and registration documents).

15. DATA HANDLING AND RECORD KEEPING

15.1 Data Handling

The Investigator will have overall responsibility for receiving, entering, cleaning, querying, analysing and storing all data that accrues from the study, but these tasks may be delegated to other Investigators. The data will be entered into the volunteers' CRFs. This includes safety data, laboratory data (both clinical and immunological) and outcome data.

15.2 Record Keeping

The investigators will maintain appropriate medical and research records for this trial, in compliance with the requirements of the Medicines for Human Use (Clinical Trial) Regulations 2004, ICH E6 GCP and regulatory and institutional requirements for the protection of confidentiality of volunteers. The Principal Investigator, co-investigators and clinical research nurses will have access to records. The investigators will permit authorized representatives of the sponsor(s), and regulatory agencies to examine (and when required by applicable law, to copy) clinical records for the purposes of quality assurance reviews, audits and evaluation of the study safety and progress.

15.3 Source Data and Case Report Forms (CRFs)

All protocol required information will be collected in CRFs designed by the investigators. Source documents are original documents, data, and records from which the volunteer's CRF data are obtained. For this study, these will include, but are not limited to, volunteer consent form, blood results, GP response letters, laboratory records, diaries, and correspondence. In the majority of cases, CRF entries will be considered source data as the CRF is the site of the original recording (i.e., there is no other written or electronic record of data). In this study this will include, but is not limited to medical history, medication records, vital signs, physical examination records, urine assessments, blood results, adverse event data and details of vaccinations. All source data and volunteer CRFs will be stored securely.

15.4 Data Protection

The study protocol, documentation, data and all other information generated will be held in strict confidence. No information concerning the study or the data will be released to any unauthorized third party, without prior written approval of the sponsor.

15.5 Use, Storage, and Tracking of Specimens and Data

Samples and data collected under this protocol will be used to study malaria and related diseases, and possible adverse reactions to vaccination. Access to research samples will be limited using either a locked room or a locked freezer. Samples and data will be stored using codes assigned by C.I.s or their designees. Data will be kept in password-protected computers. Only investigators or their designees will have access to the samples and data.

Samples stored at the MVDB in Rockville, MD will be tracked using a sample tracking software program, e.g. Freezerworks. The research use of stored, unlinked, or unidentified samples may be exempt from the need for prospective IRB review and approval. Exemption requests will be submitted in writing to the NIH Office of Human Subjects Research, which is authorized to determine whether a research activity is exempt. Samples may be stored at the CCVTM for up to 15 years.

Any loss or unanticipated destruction of samples (for example, due to freezer malfunction) or data (for example, misplacing a printout of data with identifiers) will be reported to the ethics committees/IRBs. Such a loss will be reported to the National Institute of Allergy and Infectious Diseases (NIAID) IRB as a protocol violation under the following classification: The violation compromises the scientific integrity of the data collected for the study.

© The University of Oxford 2010

Subjects may decide at any point not to have their samples stored. In this case, the C.I. or the MVDB will destroy all known remaining samples and report this to both the subject and to the IRBs. This decision will not affect the subject's participation in this trial.

16. FINANCING AND INSURANCE

16.1 Compensation / Expenses

Volunteers will be compensated for their time and travel and for study procedures while participating in the study as detailed below:

**Pre-screening visit:**	£25
**Vaccination Group:**	
Travel (40) @ £6	£252
Venepuncture (40) @ £6	£240
Time in clinic (34.5hrs) @ £15/hr	£517.50
Malaria convalescence (3days)	£1080
Unscheduled repeat visits/Venepunucture	£98
**Total**	**£2187.50**
**Control group:**	
Travel (32) @ £6	£192
Venepuncture (34) @ £6	£204
Time in clinic (23.5hrs) @ £15/hr	£352.5
Malaria convalescence (3days)	£1080
Unscheduled repeat visits/Venepunucture	£74
**Total**	**£1902.5**

Alternates will be given a sum of £60 if they are not enrolled

16.2 Insurance

Oxford University Investigators participating in this trial will receive insurance coverage from the University clinical trials insurance policy. Volunteers will have access to full NHS care if required. Compensation for any injury caused by taking part in this study will be in accordance with the guidelines of the Association of the British Pharmaceutical Industry (ABPI) under the University's Clinical Trials insurance policy.

17. PUBLICATION POLICY

The Investigators will co-ordinate dissemination of data from this study. All publications (e.g., manuscripts, abstracts, oral/slide presentations, book chapters) based on this study will be submitted to co-authors and laboratory chiefs for review before release, as per the policy of the Investigators' institutions.

© The University of Oxford 2010 **CONFIDENTIAL**

APPENDIX A: SCHEDULE OF ATTENDANCES

Attendance ref.	PS	S	V1	V1+3	V1+7	V1+14	V1+28	V2	V2+3	V2+7	V2+14, C	C+1	C+2-12	C+13-16	C+28	C+70	C+168
**Attendance number (vaccination group)**	**1**	**2**	**3**	**4**	**5**	**6**	**7**	**8**	**9**	**10**	**11**	**12**	**13-34**	**35-38**	**39**	**40**	**41**
**Attendance number (control group)**	**1**	**2**									**3**	**4**	**5-26**	**27-30**	**31**	**32**	**33**
**Timeline (days)**	PS	S	D0	D3	D7	D14	D28	D56	D59	D63	D70	D71	D72-82 (AM+PM)	D83-D86	D98	D140	D238
**Time windows (days)**	±90	±90		±1	±2	±3		±3	±1	±2	±3				±7		±14
**Vaccination**			X					X									
**Malaria Challenge**											X						
**Inclusion / Exclusion criteria**		X															
**Informed consent**		X															
**Medical history**		X															
**Physical examination**		X															X
**Urinalysis**		X	X		X	X		X		X	X				X		X
**β-HCG urine test**		X	X			X		X			X						X
**Review contraindications**		X	X					X			X						
**Vital signs**		X	X	X	X	X	X	X	X	X	X	X	X	X	X	X	X
**Local & systemic events/reactions**			X	X	X	X	X	X	X	X	X	X	X	X	X	X	X
**Diary cards provided**			1					2									
**Diary cards collected**					1					2							
**HLA typing (mL)****			3								**						
**EBV, CMV (mL)**	5*																
**HBV, HCV, HIV (mL)**		0*															5
**Anti-dsDNA (mL)**		5					5				5						5
**Biochemistry (mL)**		4	4		4	4	4	4		4	4				4	4	4
**Haematology (mL)**		2	2		2	2	2	2		2	2				2	2	2
**Serum storage (mL)**			5														
**Serum ELISA (mL)**			5		5	5	5	5		5	5			5	5	5	5
**Exploratory immunology (mL)**			60			60		60			60				60		60
**Serum GIA (mL)**			15								15				15		15
**Thick film smear / PCR (mL)**												2	2 x 22	2 x 4			
**Blood volume (mL)**	5	11	94	0	11	71	16	71	0	11	91	2	44	13	86	11	96
**Cumulative blood volume (mL) Vaccination Group**	**5**	**16**	**110**		**121**	**192**	**208**	**279**		**290**	**381**	**383**	**427**	**440**	**526**	**537**	**633**
**Cumulative blood volume (mL) Control Group**	**5**	**16**									**110**	**112**	**156**	**169**	**255**	**266**	**362**

1. Timeline is approximate only. Exact timings of visits relate to the previous visit – *i.e.* each visit must fall within the specified number of days after the last visit ± time window.
2. S = Screening; V = Vaccination; C = Challenge. Columns highlight vaccination/challenge days.
3. Volunteers will also be required to attend for visits 1 and 2 days after treatment for malaria (not shown on schedule).
* Serology for HIV, Hep B & Hep C will be added to the sample taken for serology at pre-screening with the volunteers consent if this is available, otherwise volunteers will donate an additional 5ml sample for HIV, HBV and HCV antibody testing.
** HLA performed at C for Group 2 volunteers

APPENDIX B: LABORATORY VALUES FOR EXCLUSION

PARAMETER	LOWER LIMIT OF EXCLUSION	UPPER LIMIT OF EXCLUSION
**BIOCHEMISTRY**		
Potassium [mmol/L]	<3.2	>5.5
Sodium [mmol/L]	<132	>148
Urea [mmol/L]	N/A	>9
Creatinine [μmol/L]	N/A	>145
Albumin [g/L]	<30	N/A
Total bilirubin [μmol/L]	N/A	>25 when accompanied by elevated liver enzyme
ALT [IU/L]	N/A	> 56
ALP [IU/L]	N/A	>350
**HAEMATOLOGY**		
Haemoglobin [g/dL]	Male: < 11.5<br>Female: < 11.0	Male: > 18<br>Female: >17.5
White Cell Count [x 10^9/L]<br>Neutrophil count [x 10^9/L]	<3.5<br>< 1.5	>14.0
Platelet Count [x 10^9/L]	<135	>500
**URINE ANALYSIS (using MULTISTIX * 10 SG Bayer Diagnostics)**		
Protein [g/L]	> 0.3	
Glucose [mmol/L]	> 5.5	

APPENDIX C: GRADING SCALES FOR ADVERSE EVENTS

Adverse Event	Grade	Intensity
Pain at injection site	1<br>2<br>3	Pain that is easily tolerated<br>Pain that interferes with daily activity<br>Pain that prevents daily activity
Erythema at injection site	1<br>2<br>3	>0 - ≤20 mm<br>>20 - ≤50 mm<br>>50 mm
Swelling at injection site	1<br>2<br>3	>0 - ≤20 mm<br>>20 - ≤50 mm<br>>50 mm
Fever (oral)	1<br>2<br>3	37.6°C - 38.0°C<br>>38.0°C – 39.0°C<br>>39.0°C
Headache	1<br>2<br>3	Headache that is easily tolerated<br>Headache that interferes with daily activity<br>Headache that prevents daily activity
Nausea	1<br>2<br>3	Nausea that is easily tolerated<br>Nausea that interferes with daily activity<br>Nausea that prevents daily activity
Malaise	1<br>2<br>3	Malaise that is easily tolerated<br>Malaise that interferes with daily activity<br>Malaise that prevents daily activity
Myalgia	1<br>2<br>3	Myalgia that is easily tolerated<br>Myalgia that interferes with daily activity<br>Myalgia that prevents daily activity
Arthralgia	1<br>2<br>3	Joint pain that is easily tolerated<br>Joint pain that interferes with daily activity<br>Joint pain that prevents daily activity
Urticaria	1<br>2<br>3	Requiring no medications<br>Requiring PO or topical treatment or IV medication or steroids for <24 hours<br>Requiring IV medication or steroids for >24 hours

APPENDIX D: TOXICITY TABLE FOR GRADING LABORATORY ADVERSE EVENTS

Laboratory Test	Grade 1	Grade 2	Grade 3	Grade 4 (Life threatening)
Hgb (female) – decrease from testing laboratory LLN in gm/dl	1.0 - <1.5	≥1.5 & <2.0	≥2.0	Requires transfusion
Hgb (male) – decrease from testing laboratory LLN in gm/dl	≥1.5 & <2.0	≥2.0 & <2.5	≥2.5	Requires transfusion
Absolute neutrophil count (ANC, cells/mm^3)	1000-1499	500-999	<500	<500 with fever
Leukopenia (WBC, cells/mm^3)	<3500 - ≥2500	<2500 - ≥1500	<1500	<1500 with fever
Platelets (cells/mm^3)	125,000 – 135,000	100,000 – 124,000	20,000-99,000	<20,000
ALT	1.25 – 2.5 x ULN	>2.6 – 5.0 x ULN	>5.0 x ULN	>10 x ULN and requires hospitalization
Creatinine	1.1 – 1.5 x ULN	>1.6 – 3.0 x ULN	>3.0 x ULN	>5.0 x ULN and requires dialysis
Urine protein	2+ or 0.5-1 gm loss/day	3+ or 1-2 gm loss/day	4+ or >2 gm loss/day	NA
Hematuria	2+ confirmed by 5-10 rbc/hpf	3+ confirmed by >10 rbc/hpf	gross, with or without clots, OR red blood cell casts	NA

ULN = Upper limits of normal

APPENDIX E: TABLE FOR GRADING ADVERSE EVENTS

These tables are to be used to grade unexpected adverse events not described in Appendices C and D.

**Vital Signs***	**Mild (Grade 1)**	**Moderate (Grade 2)**	**Severe (Grade 3)**	**Life threatening (Grade 4)**
Tachycardia – beats per minute	110-120	120-140	>140	>160 with symptoms**
Bradycardia – beats per minute (if associated with symptoms***)	50-54	45-49	<45	<35 with loss of consciousness
Hypertension (systolic) – mm Hg (with repeat testing at same visit)	141-150	151-170	>170	>200 with symptoms**
Hypertension (diastolic) – mm Hg (with repeat testing at same visit)	91-100	100-110	>110	>120 with symptoms**
Hypotension (systolic) – mm Hg (with repeat testing at same visit)	85-89 (and symptomatic)	80-84 (and symptomatic)	<80	<70 with loss of consciousness

* Participant should be at rest for measurement of vital signs
** Severe headache, chest pain, altered consciousness, or other signs of acute organ damage
*** Lightheadedness

**Systemic**	**Mild (Grade 1)**	**Moderate (Grade 2)**	**Severe (Grade 3)**	**Life Threatening (Grade 4)**
Anorexia	Loss of appetite without decreased oral intake lasting greater than 48 hours	Loss of appetite associated with decreased oral intake without significant weight loss	Loss of appetite associated with significant weight loss	NA
Vomiting	1-2 episodes/24 hours	>2 episodes/24 hours	Prevents daily activity, requires outpatient IV hydration	Requires hospitalization
Diarrhea	2-3 loose stools/24 hours	4-5 loose stools/24 hours	>6 loose stools or requires outpatient IV hydration	Requires hospitalization
Constipation	NA	Persistent constipation requiring regular use of dietary modifications, laxatives, or enemas	Obstipation with manual evacuation indicated	NA
Fatigue	No interference w/activity	Some interference w/activity	Significant, prevents daily activity	NA

Arthritis	Mild pain with inflammation, erythema or joint swelling – but not interfering with function	Moderate pain with inflammation, erythema or joint swelling – interfering with function, but not with activities of daily living	Severe pain with inflammation, erythema or joint swelling –and interfering with activities of daily living	NA
Mucocutaneous Reaction/Rash	Erythema; pruritis or localized macular rash	Diffuse, maculo-papular rash, dry desquamation	Vesiculation or moist desquamation or ulceration	Requires hospitalization
Vasovagal episode (associated with a procedure of any kind)	Present without loss of consciousness	Present with transient loss of consciousness	NA	NA
Vertigo	Causes no or minimal interference with usual daily activities	Causes greater than minimal interference with usual daily activities	Inability to perform daily activities	NA
Cough	transient- no treatment	persistent cough; treatment responsive	Paroxysmal cough; uncontrolled with treatment	Requires hospitalization
Bronchospasm, Acute	transient; no treatment; 70% - 80% FEV_1 of peak flow	requires treatment; normalizes with bronchodilator; FEV_1 50% - 70% (of peak flow)	no normalization with bronchodilator; FEV_1 25% - 50% of peak flow; or retractions present	Requires hospitalization
Dyspnea	Dyspnea on exertion	Dyspnea with normal activity	Dyspnea at rest	Requires hospitalization

For AEs not otherwise described severity will be graded as follows:

GRADE 1 Mild: no effect on activities of daily living; no medical intervention/therapy required

GRADE 2 Moderate: partial limitation in activities of daily living (can complete >50% of baseline); no or minimal medical intervention/therapy required

GRADE 3 Severe: activities of daily living limited to <50% of baseline; medical evaluation/therapy required

GRADE 4 Life Threatening

18. REFERENCES

Anders, R. F., Crewther, P. E., Edwards, S., Margetts, M., Matthew, M. L., Pollock, B. and Pye, D. (1998). Immunisation with recombinant AMA-1 protects mice against infection with Plasmodium chabaudi. *Vaccine* **16**, 240-7.

Bejon, P., Andrews, L., Andersen, R. F., Dunachie, S., Webster, D., Walther, M., Gilbert, S. C., Peto, T. and Hill, A. V. (2005). Calculation of Liver-to-Blood Inocula, Parasite Growth Rates, and Preerythrocytic Vaccine Efficacy, from Serial Quantitative Polymerase Chain Reaction Studies of Volunteers Challenged with Malaria Sporozoites. *J Infect Dis* **191**, 619-26.

Cheng, Q., Lawrence, G., Reed, C., Stowers, A., Ranford-Cartwright, L., Creasey, A., Carter, R., Saul, A. (1997). Measurement of Plasmodium falciparum growth rates in vivo: a test of malaria vaccines*. Am J Trop Med Hyg* **57,** 495-500.

Conroy, R.M., **Pyörälä, K**., **Fitzgerald, A.P**., **Sans, S**, **Menotti, A**., **De Backer, G**., **De Bacquer, D**., **Ducimetière, P**., **Jousilahti, P**., **Keil, U**., **Njølstad, I**., **Oganov, R.G**., **Thomsen, T**., **Tunstall-Pedoe, H**., **Tverdal, A**., **Wedel, H**., **Whincup, P**., **Wilhelmsen, L**., **Graham, I.M**. (2003). Estimation of ten-year risk of fatal cardiovascular disease in Europe: the SCORE project. *Eur Heart J* **24**(11), 987-1003.

Draper, S. J., Moore, A. C., Gilbert, S. C., Hill, F. and Hill, A. V. S. (2007). Multi-stage protection against murine malaria using recombinant viral vaccines expressing merozoite surface protein-1. *Submitted*.

Malkin, E. M., Diemert, D. J., McArthur, J. H., Perreault, J. R., Miles, A. P., Giersing, B. K., Mullen, G. E., Orcutt, A., Muratova, O., Awkal, M. et al. (2005). Phase 1 clinical trial of apical membrane antigen 1: an asexual blood-stage vaccine for Plasmodium falciparum malaria. *Infect Immun* **73**, 3677-85.

Miao, J., Li, X., Liu, Z., Xue, C., Bujard, H., Cui, L. (2006). Immune responses in mice induced by prime-boost schemes of the Plasmodium falciparum apical membrane antigen 1 (PfAMA1)-based DNA, protein and recombinant modified vaccinia Ankara vaccines. *Vaccine* **24,** 6187-98

Mueller, M. S., Renard, A., Boato, F., Vogel, D., Naegeli, M., Zurbriggen, R., Robinson, J. A. and Pluschke, G. (2003). Induction of parasite growth-inhibitory antibodies by a virosomal formulation of a peptidomimetic of loop I from domain III of Plasmodium falciparum apical membrane antigen 1. *Infect Immun* **71**, 4749-58.

Narum, D. L., Ogun, S. A.,Thomas, A. W.,Holder, A. A.(2000). Immunization with parasite-derived apical membrane antigen 1 or passive immunization with a specific monoclonal antibody protects BALB/c mice against lethal Plasmodium yoelii yoelii YM blood-stage infection. *Infec Immun* 68, 2899-906

Polley, S. D. and Conway, D. J. (2001). Strong diversifying selection on domains of the Plasmodium falciparum apical membrane antigen 1 gene. *Genetics* **158**, 1505-12.

Polley, S. D., Mwangi, T., Kocken, C. H., Thomas, A. W., Dutta, S., Lanar, D. E., Remarque, E., Ross, A., Williams, T. N., Mwambingu, G. et al. (2004). Human antibodies to recombinant protein constructs of Plasmodium falciparum Apical Membrane Antigen 1 (AMA1) and their associations with protection from malaria. *Vaccine* **23**, 718-28.

Rogers, W. O., Weiss, W. R., Kumar, A., Aguiar, J. C., Tine, J. A., Gwadz, R., Harre, J. G., Gowda, K., Rathore, D., Kumar, S. et al. (2002). Protection of rhesus macaques against lethal Plasmodium knowlesi malaria by a heterologous DNA priming and poxvirus boosting immunization regimen. *Infect Immun* **70**, 4329-35.

Silvie, O., Franetich, J. F., Charrin, S., Mueller, M. S., Siau, A., Bodescot, M., Rubinstein, E., Hannoun, L., Charoenvit, Y., Kocken, C. H. et al. (2004). A role for apical

membrane antigen 1 during invasion of hepatocytes by Plasmodium falciparum sporozoites. *J Biol Chem* **279**, 9490-6.

Stowers, A. W., Kennedy, M. C., Keegan, B. P., Saul, A., Long, C. A. and Miller, L. H. (2002). Vaccination of monkeys with recombinant Plasmodium falciparum apical membrane antigen 1 confers protection against blood-stage malaria. *Infect Immun* **70**, 6961-7.

van Vugt, M., Looareesuwan, S., Wilairatana, P., McGready, R., Villegas, L., Gathmann, I., Mull, R., Brockman, A., White, N. J., Nosten, F. (2000). Artemether-lumefantrine for the treatment of multidrug-resistant falciparum malaria. *Trans R Soc Trop Med Hyg* **94. 545-8**

WHO. (2002). World Health Organisation Expert Committee on Malaria 20th Report, (ed. Geneva: WHO.
